# Supplementary material for: Single-molecule level control of host-guest interactions in metallocycle-C60 complexes
Source: Nat Commun. 2019 Oct 10;10:4599. doi: 10.1038/s41467-019-12534-6 (PMC6787074; doi:10.1038/s41467-019-12534-6)
Supplement: Supplementary file 1 — Supplementary Information [file 41467_2019_12534_MOESM1_ESM.pdf]

Reviewers' Comments:

Reviewer #1:
Remarks to the Author:
Report on Nature Communications manuscript NCOMMS 18 37806 T

The field of molecular electronics was revived by the development of reliable methods for making and characterizing metal-single molecule-metal junctions about 15 years ago (including a scanning probe microscopy-based method pioneered by one of the corresponding authors of this submission). Since then, the science of single molecule junction electrical, thermal and spintronic properties has advanced greatly. Probing the single molecule behavior of supramolecular species is an avenue that is ripe for exploration and is now developing. In this submission, the electrical properties of junctions with some supramolecular Pt organometallic assemblies and their host-guest complexes with C60 are examined. The authors claim that on junction extension, strain causes the molecule:C60 complexes to dissociate, resulting in a conductance decrease. This would be suitable for publication in a general interest journal such as Nature Communications if the data were suitably strong. Unfortunately there are some doubts that this is so, and publication is not recommended in the present form, because of the weaknesses identified below.

The supramolecular coordination complexes are well-characterized. Their interaction with C60 in solution are examined; molecule 5 with the smallest cavity does not significantly interact with C60 in solution because there is no perturbation of the electronic spectrum indicative of a charge transfer interaction, but complex 6 does interact because a new intermolecular charge transfer band appears in the spectrum; the formation constant is high (over 105). Complex 7 in contrast is too large and again, with C60 there is no significant interaction in solution.

The first issue is that the molecules are all coordination complexes of Pt(II) with aryl ligands, phosphines, and pyridines. The latter in particular might represent a weak point when such molecules interact with a bare gold surface. For a paper making a high impact claim like this, some independent evidence that the molecules remain intact upon adsorption to gold surfaces should be obtained (e.g. surface vibrational spectroscopy, XPS and/or NEXAFS etc.) so that one can be confident that junctions are indeed forming with intact molecules and that the higher conductance values are indeed due to C60 complexes. The break-off distances (q.v.) do not seem consistent with the likely full length of the molecules (although this could well be due to them failing to be fully lifted in the junction).

The very large size of the molecules, and the fact that they all contain two meta-oriented contact groups, with resulting destructive quantum interference, means that low conductance values are expected, as is observed. It is claimed that the conductance increases by an order of magnitude or so on C60 exposure for molecules 5 and 6, but not for 7. Moreover, on junction extension, molecules 5 and 6 often revert to the low-conductance values characteristic of the free molecules, particularly at longer tip retraction distances.

The claim that molecular strain on junction extension causes C60 dissociation is potentially the most interesting observation in the paper, but to justify this significant claim, stronger evidence is needed. Some ‘pull and hold’ experiments might help here; does the complex persist in the high conductance state when the junction is not strained by pulling, just maintained at constant tip height in the junction, or does it in fact stochastically switch (as might be the case if, for instance, the C60 is being exchanged with the neighboring gold surface)? In that respect it is worth noting that, although the authors apparently found no evidence of junction formation using gold decorated with C60 alone, other workers have found that C60 interacts strongly with Au, forming self-assembled monolayers from solution (Yoshimoto et al., Langmuir 2002 18, 8518), and moreover, single molecule electrical

measurements (conductance and thermopower) have been made on C60-functionalized molecules in which it has been employed as a contact group (CA Martin et al, JACS 2008, 130, 13198; E Leary et al, Nano Letts. 2011, 11, 2236; SK Lee et al Nano Letts. 2014, 14, 5276). It appears surprising that although molecule 5 does not interact strongly enough with C60 in solution to perturb the electronic spectrum, it does experience a conductance increase when its monolayer on Au is interacted with C60. This appears surprising and some discussion of why this should occur is warranted.

Incidentally, how is the y-axis in Figure 4(a-c) calibrated? The junction extensions of only ca. 0.4 nm seem to suggest that, even allowing for the usual snap-back distance, the molecules are not in fact being fully lifted in the junction (contrary to the cartoon in Fig. 2). Is it not possible that the conductance increase is indeed due to C60 interacting in some way with the molecule in the junction (either in the cavity for 6, or more peripherally for 5), but as the molecule is lifted, the C60 adheres strongly to the gold surface and it is actually this which causes the junctions to break down?

In the theory section of the paper, T(E) curves are used to support the notion of (i) conductance increase on complexation and (ii) conductance decrease on molecular distortion. How well (or otherwise) do the theoretical conductance values for the molecules and their complexes correlate with experiment?

The complex between 6 and C60 is clearly a charge transfer interaction from the electronic spectroscopy data, so: does this result in a Fano resonance near the contact Fermi energy as seen by another of the corresponding authors Lambert (et al) in their recent paper on oligothiophene-TCNE charge transfer complexes (Nanoscale 2015, 7, 18949)? It is hard to tell from the Figures, as these are pyridine-contacted molecules and there is the usual proximity of the LUMO resonances to E(F) seen with this contact group, although there does seem to be an additional peak for the complex with 6. (Incidentally, why is the latter paper not referenced? It would seem to be relevant here).

In summary, this might be a suitable paper for resubmission to Nature Communications with (i) stronger evidence in favor of the mechanism suggested by the authors (ii) additional surface characterization of the molecular monolayers.

Reviewer #2:
Remarks to the Author:
There has been an increasing level of interest in determining the single molecular conductance of increasingly complex molecular and biomolecular structures and this has included structures that exploit weaker non-covalent interactions, with some of these studies being referenced (references 28-32). This present work extends such investigations to an interesting and chemically rather exquisite supramolecular assembly which involves the insertion of C60. Tang et al show here that conductance of organoplatinum(II) metallocycle hosts can be significant enhanced by the incorporation of a fullerene guest molecule. This is shown using the STM- break junction method (STM-BJ), a very highly cited and respected method for molecular conductance determination which was invented by one of the co-authors (Nongjan Tao) and Bingqian Xu. Conceptually new here is the demonstration that mechanically stretching the metallocycle-C60 junction with this technique can induce the expulsion of the fullerene guest from the metallocycle. This then provides a form of mechanical conductance switch.
I support publication but would recommend some additions to underpin the STM measurements section as listed below:
(1) The authors should demonstrate that the junction breaking distances are comparable for the with and without C60 cases. I can believe that the junction is fully elongated with the C60 in place as in the

cartoon in Figure 2 (top right) but this may not be the case with the empty macrocycles which contain a plethora of possible STM tip attachment points and can also tilt towards the surfaces. This can be straightforwardly resolved by presenting conventional 2D histograms of junction conductance versus stretching distance to show that the empty macrocycle is placed at its break off point as shown the cartoon in Figure 2 (top left).
(2) I would also suggest presenting the 1 (and 2D mentioned above) histograms to show the G(0) peaks from the break junction breaking since this reinforces the data quality.
(3) On page 5 can the implications of the following statement for the STM-BJ results be further clarified "The failure to observe potential CT absorption band from the complexation of 5 with C60 is possibly caused by the relatively weak and shallow interaction between them. This is supported by the DFT calculation results discussed below." If the interaction is weak and shallow why is the host-guest complexation peak seen in figure 3d as just a large peak as for 6 + C60 in Figure 3e?

Reviewer #3:
Remarks to the Author:
This paper reports on the single-molecule conductance of a host-guest complex. Organoplatnium metallocycles are synthesized and their conductance measured with the STMBJ technique. The molecular conductance can be increased by one order of magnitude when a C60 molecule is encapsulated. Conductance-distance histograms for two metallocycles show two plateaus, which are interpreted as corresponding to the free host (low conductance) and host-C60 complex (high conductance). When increasing the tip distance the C60 molecule is released and the conductance of the free host is recovered. Theoretical calculations aid in the interpretation of measurements. The results are compelling and add to a growing number of papers investigating conductance of host-guest complexes. I recommend publication but some issues need to be addressed first.

The increase in conductance due to C60 seems convincing in view of the STMBJ histograms of figure 3. New conductance peaks are observed for the [5+C60] and [6+C60] complexes. The large cavity size of 7 suggests no sufficient host-guest interaction. The paper mentions mechanical control of host-guest interactions as an attractive strategy however the conductance switch described by the authors seems to take place in one direction only (release of C60). Can encapsulation be induced?

Control experiments with C60 solution showed no conductance peak, contrary to reports in the literature for this molecule, why?

Theoretical calculations demonstrate the potential to modulate host-guest interactions by changing host size. Transmission calculations show that C60 increases the conductance of the host providing an extra current pathway. Theoretical conductance is smaller than experimental values, in contradiction to the results of DFT calculations in the literature. This should have been discussed but was not mentioned. The enhancement of conductance by the C60 host is expected and is demonstrated by the calculations. However, there seems to be a fundamental disagreement between calculations and experiment where calculations show less conductance, not more.

The effect of donor-acceptor interactions in the metallocycle-C60 calculations needs some clarification. Host-guest charge transfer shifts the frontier orbitals of C60 (eg. through the intermediate configurations in figure 6). The shift in C60 HOMO is very large however the LUMO is unchanged. The LUMO of the C60 acceptor in the host-guest is not filled, why not?

A 100 times increase in transmission was obtained when the Au-N bond was almost perpendicular to the plane of the pyridine ring. Although intriguing, without any additional information it is not possible

to judge whether this geometry is in any way relevant or energetically favorable. Is such a change in conductance observed in the STMBJ experiments?

Further analysis and discussion are necessary.

Reviewer# 1

The field of molecular electronics was revived by the development of reliable methods for making and characterizing metal-single molecule-metal junctions about 15 years ago (including a scanning probe microscopy-based method pioneered by one of the corresponding authors of this submission). Since then, the science of single molecule junction electrical, thermal and spintronic properties has advanced greatly. Probing the single molecule behavior of supramolecular species is an avenue that is ripe for exploration and is now developing. In this submission, the electrical properties of junctions with some supramolecular Pt organometallic assemblies and their host-guest complexes with C60 are examined. The authors claim that on junction extension, strain causes the molecule: C60 complexes to dissociate, resulting in a conductance decrease. This would be suitable for publication in a general interest journal such as Nature Communications if the data were suitably strong. Unfortunately there are some doubts that this is so, and publication is not recommended in the present form, because of the weaknesses identified below.

The supramolecular coordination complexes are well-characterized. Their interaction with C60 in solution are examined; molecule 5 with the smallest cavity does not significantly interact with C60 in solution because there is no perturbation of the electronic spectrum indicative of a charge transfer interaction, but complex 6 does interact because a new intermolecular charge transfer band appears in the spectrum; the formation constant is high (over 105). Complex 7 in contrast is too large and again, with C60 there is no significant interaction in solution.

The first issue is that the molecules are all coordination complexes of Pt(II) with aryl ligands, phosphines, and pyridines. The latter in particular might represent a weak point when such molecules interact with a bare gold surface. For a paper making a high impact claim like this, some independent evidence that the molecules remain intact upon adsorption to gold surfaces should be obtained (e.g. surface vibrational spectroscopy, XPS and/or NEXAFS etc.) so that one can be confident that junctions are indeed forming with intact molecules and that the higher conductance values are indeed due to C60 complexes.

1. Reply: We thank the reviewer for these constructive suggestions, and have performed XPS and AFM experiments to prove that the metallocycles remain intact on Au surfaces. XPS results of metallocycle monolayers on Au surfaces agree well with those of metallocycle powders (shown in Table. 1-2, Figs. 1-4, and as added to SI in Table. S1-S2, Figs. S24-S27), suggesting that the metallocycles are intact after they are absorbed onto Au surfaces. Moreover, the height changes of the resulting films are consistent with the size of the metallocycles in AFM images (Fig. 5, added to SI as Fig. S28), providing further evidences of the formation of metallocycles functionalized Au surfaces. We have discussed these results in the main article as follows:

XPS characterizations. To prove that the metallocycles remain intact on Au surfaces, XPS characterizations were carried out on monolayers on Au surfaces and corresponding powders of **5** and **6**. As shown in Figs. S24-S27, the selected signals of Pt4f, P2p, N1s and F1s are observed in both powders and monolayers. According to the further quantitative analysis (Tabs. S1-S2), the atomic percentages of the elements of monolayers agree well with the powders compositions. For example, the Pt/P/N/F atomic ratio of **5** monolayer (1.0/1.4/3.0/2.6) is consistent with that of the powder sample (1.0/2.1/2.7/2.7) within experimental error.

AFM imaging. AFM images of the bare Au surface and those modified with **5**, [**5** + C_{60}], **6**, [**6** + C_{60}] are shown in Fig. S28, with the root mean square roughness (rms) of 4.51, 1.29, 0.69, 0.70 and 0.55 nm, respectively. This suggests that the Au surface was slightly smoothed after being functionalized with metallocycles and metallocycle-C_{60} complexes. The height changes of the films are in accordance with the size of metallocycles. Moreover, after the

incorporation of C_{60}, the films of the [**5** + C_{60}] and [**6** + C_{60}] complexes exhibit almost the same height changes relative to corresponding free metallocycles. These results not only suggest a perpendicular configuration of metallocycles but also provide insights into the formation of host-guest complexes on Au surfaces.

Table 1. XPS survey of **5**.

atomic %	**5**	
	on Au	powder
Pt4f	0.89	0.75
P2p	1.27	1.60
N1s	2.65	2.01
F1s	2.31	2.03
atomic ratio of (Pt/P/N/F)	1.0/1.4/3.0/2.6	1.0/2.1/2.7/2.7

Table 2. XPS survey of **6**.

atomic %	**6**	
	on Au	powder
Pt4f	0.78	0.75
P2p	1.07	1.50
N1s	3.00	2.36
F1s	2.17	3.73
atomic ratio of (Pt/P/N/F)	1.0/1.4/3.8/2.8	1.0/2.0/3.1/5.0

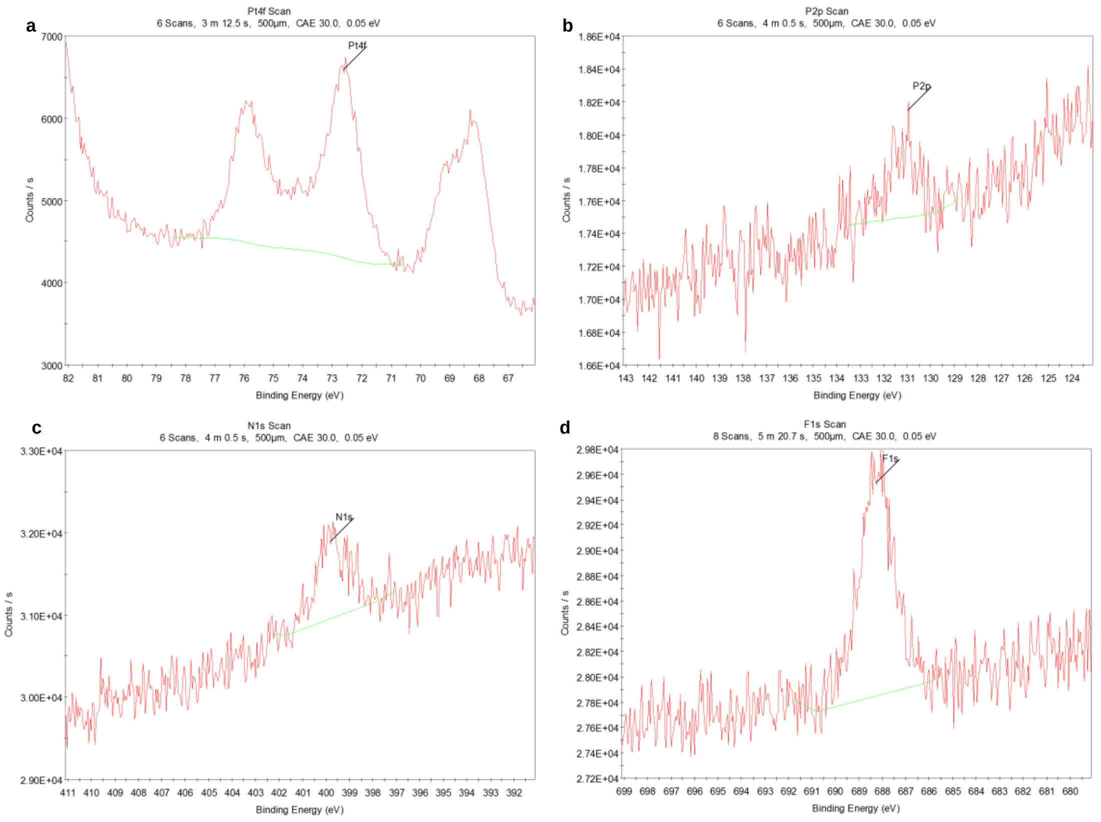

Fig. 1. Pt4f, P2p, N1s and F1s XPS spectra of **5** on Au.

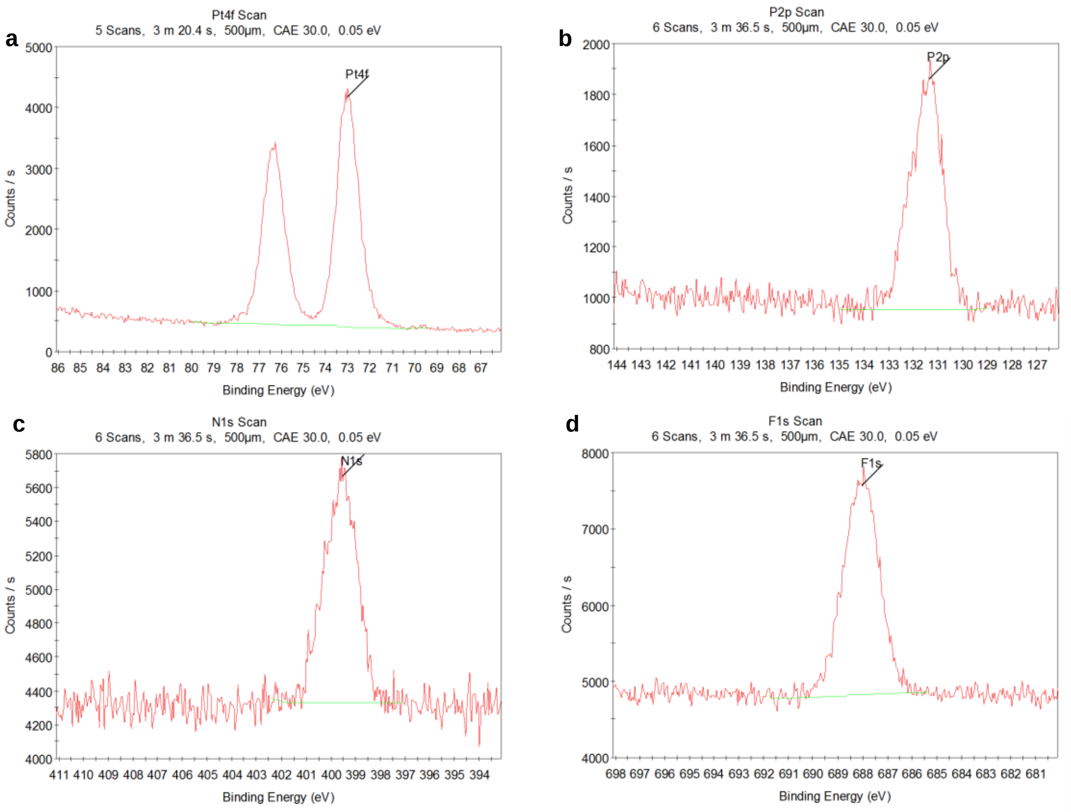

Fig. 2. Pt4f, P2p, N1s and F1s XPS spectra of the powder of **5**.

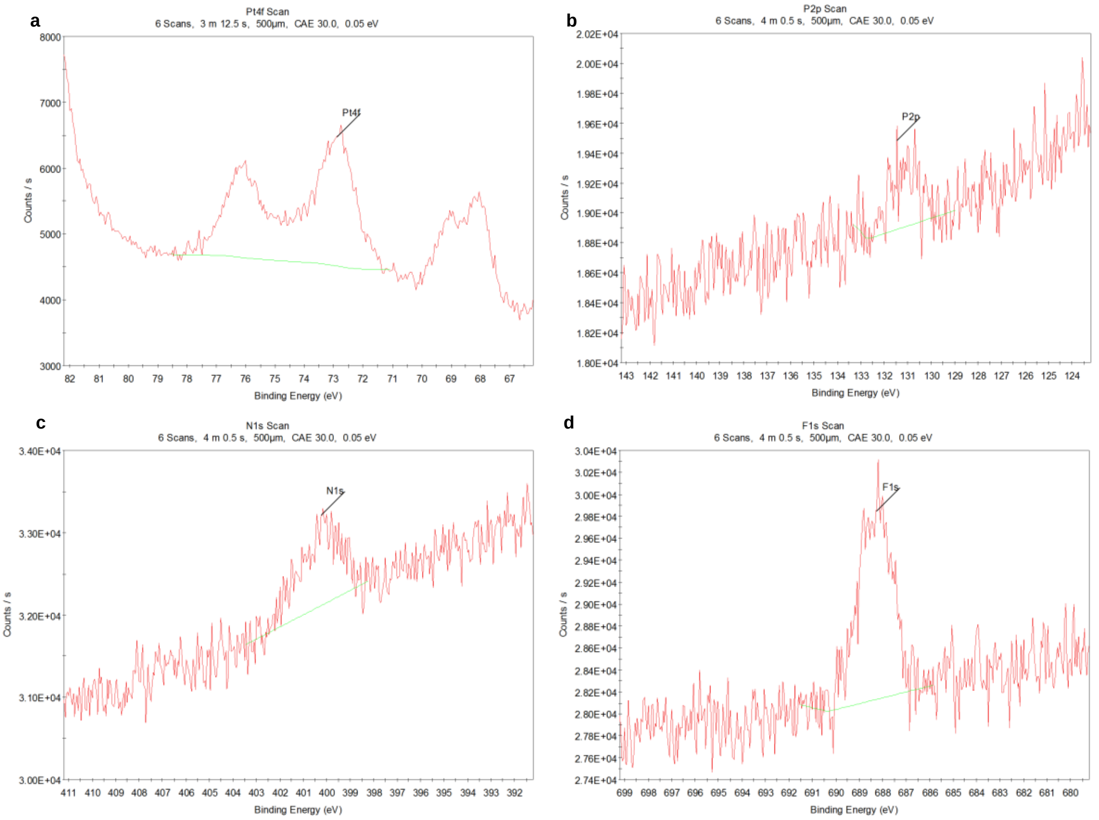

Fig. 3. Pt4f, P2p, N1s and F1s XPS spectra of **6** on Au.

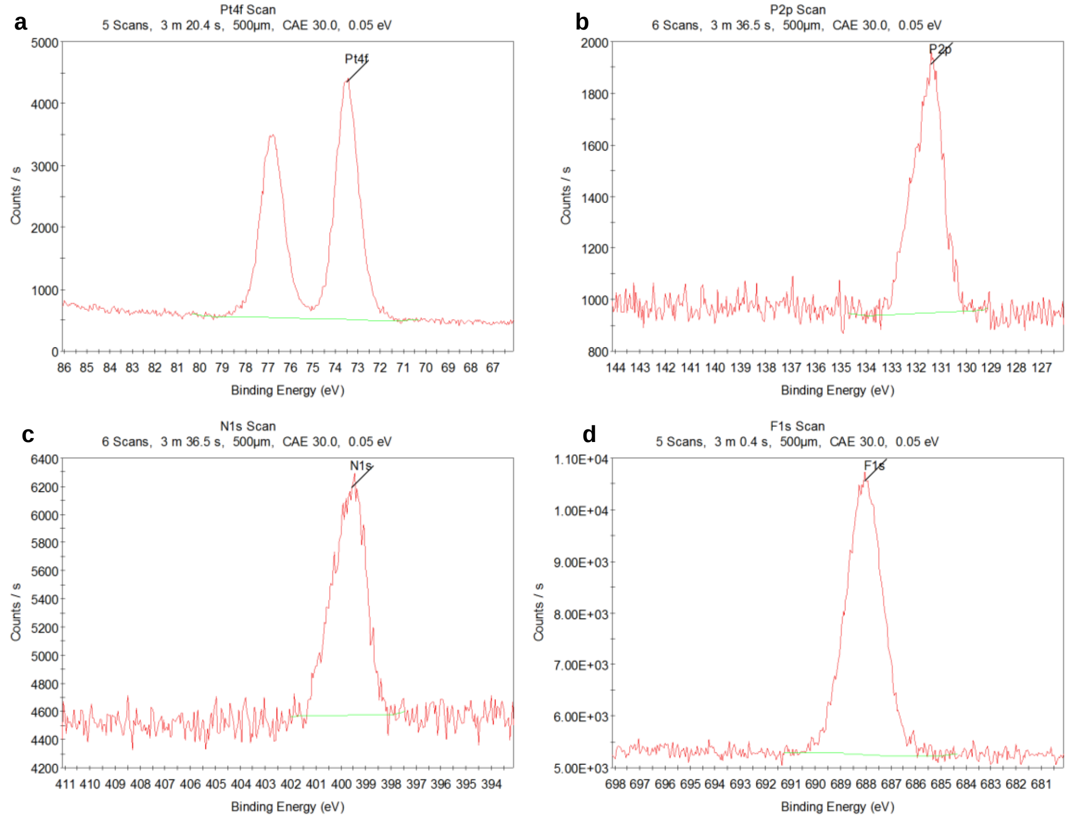

Fig. 4. Pt4f, P2p, N1s and F1s XPS spectra of the powder of **6**.

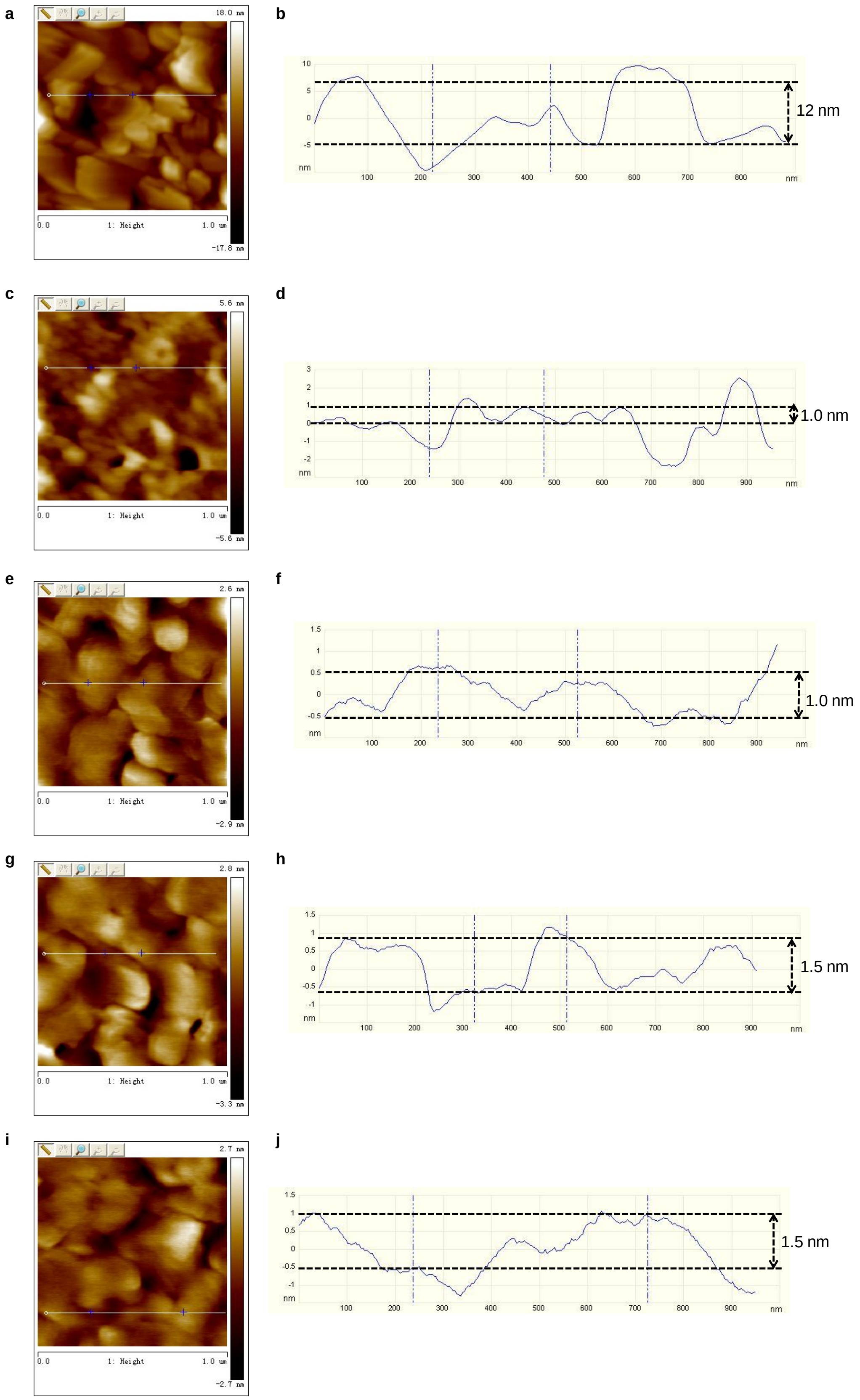

Fig. 5. AFM images and corresponding height changes of bare Au (a, b), Au/**5** (c, d), Au/[**5** + C_{60}] (e, f), Au/**6** (g, h) and Au/[**6** + C_{60}] (i, j).

The break-off distances (q.v.) do not seem consistent with the likely full length of the molecules (although this could well be due to them failing to be fully lifted in the junction).

2. Reply: In this experiment, a linear voltage amplifier was used because of its high accuracy, but its conductance range is limited to ~4 orders of magnitude and gold quantum point contact conductance (G_0) falls out of the measurable range of the linear amplifier. To obtain the most accurate conductance measurement with the setup, we thus adjusted the amplifier gain and bias voltage such that the current in the metallocycles and their host-guest complexes is in the middle of the amplifier range ($1.4\times10^{-7}G_0$ ~$1.4\times10^{-3}G_0$ for **5** and **6**, and $7\times10^{-8}G_0$ ~$7\times10^{-4}G_0$ for **7)**. To study the configuration of metallocycles or host-guest complexes on Au surface, we performed AFM analysis as stated. The AFM analysis shows the preferable perpendicular geometry of the metallocycles and their host-guest complexes.

The very large size of the molecules, and the fact that they all contain two meta-oriented contact groups, with resulting destructive quantum interference, means that low conductance values are expected, as is observed. It is claimed that the conductance increases by an order of magnitude or so on C60 exposure for molecules 5 and 6, but not for 7. Moreover, on junction extension, molecules 5 and 6 often revert to the low-conductance values characteristic of the free molecules, particularly at longer tip retraction distances.

The claim that molecular strain on junction extension causes C60 dissociation is potentially the most interesting observation in the paper, but to justify this significant claim, stronger evidence is needed. Some 'pull and hold' experiments might help here; does the complex persist in the high conductance state when the junction is not strained by pulling, just maintained at constant tip height in the junction, or does it in fact stochastically switch (as might be the case if, for instance, the C60 is being exchanged with the neighboring gold surface)? In that respect it is worth noting that, although the authors apparently found no evidence of junction formation using gold decorated with C60 alone, other workers have found that C60 interacts strongly with Au, forming self-assembled monolayers from solution (Yoshimoto et al., Langmuir 2002 18, 8518), and moreover, single molecule electrical measurements (conductance and thermopower) have been made on C60-functionalized molecules in which it has been employed as a contact group (CA Martin et al, JACS 2008, 130, 13198; E Leary et al, Nano Letts. 2011, 11, 2236; SK Lee et al Nano Letts. 2014, 14, 5276). It appears surprising that although molecule 5 does not interact strongly enough with C60 in solution to perturb the electronic spectrum, it does experience a conductance increase when its monolayer on Au is interacted with C60. This appears surprising and some discussion of why this should occur is warranted.

3.1 Reply: We have performed the suggested 'pull and hold' experiments for [**5** + C_{60}] and [**6** + C_{60}] complexes. As shown in Fig. 6 (added to SI in Fig. S17), the higher conductance states of [**5** + C_{60}] and [**6** + C_{60}] last for more than 0.1 s and 0.2 s, respectively. This is different from the conventional break junction measurements of these complexes, where the high conductance states last for less than 0.03 s (0.59 nm at 19.5 nm/s retraction rate). These results suggest that the conductance switching from higher to lower level is mechanically dependent. We have added the following description to the revised manuscript:

'Pull and hold' experiments were also performed for [**5** + C_{60}] and [**6** + C_{60}] complexes. As demonstrated in Fig. S17, the higher conductance states of [**5** + C_{60}] and [**6** + C_{60}] last for more than 0.1 s and 0.2 s, respectively. This is different with the conventional break junction measurements of these complexes, where the high conductance states last for less than 0.03 s. These results suggest that the conductance switching from higher to lower level is mechanically dependent.

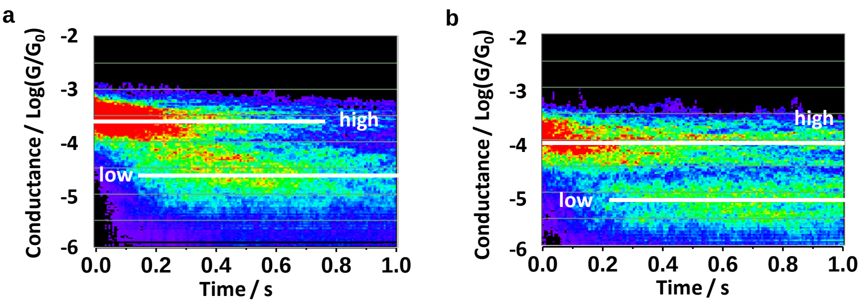

Fig. 6. 'Pull and hold' experiments of [**5** + C_{60}] (a) and [**6** + C_{60}] (b). The white horizontal lines indicate the higher and lower conductance levels. In the break junction measurement, the tip movement was halted for 1 s once a junction formation is detected.

3.2 Reply: We apologize for the typo in main text. The concentration of C_{60} in our experiments is 10 M (which was correctly described in Methods section). We agree with the reviewer that a discussion is necessary for the absence of analytical features of the conductance measurement of C_{60} modified Au surface. We emphasize that the concentration of C_{60} in our experiments was low (10 M), and after rinsing with copious amounts of solvent, the coverage of C_{60} on Au surface was too low to be measured by break junction methods (ref as in 45-50). To verify the assumption, we modified the Au surface using a higher concentration of C_{60} solution (7 mM), and removed the excess C_{60} by transferring the surface to 1,1,2,2-tetrachloroethane shortly (ref 48). Fig. 7 (added to SI as Fig. S19) shows the conductance histogram of C_{60} modified on Au surface, featured by two conductance peaks at around 0.2 G_0 and 0.5 G_0, which is in agreement with the reported values (ref 45, 49).

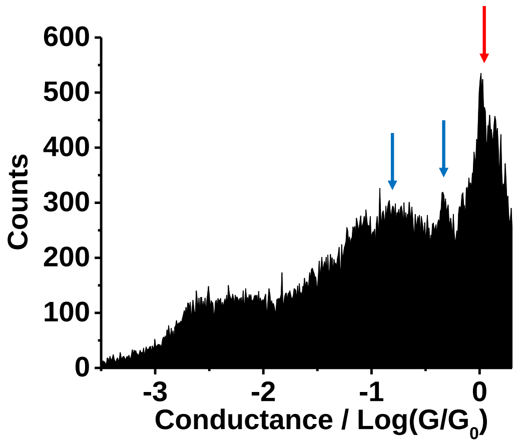

Fig. 7. Conductance histogram of C_{60}, where the Au substrate was incubated with C_{60} (7 mM) in 1,1,2,2-tetrachloroethane for 2 hours, and transferred to pure 1,1,2,2-tetrachloroethane for 1 minute, then rinsed with DI water. The blue arrows mark the conductance peaks from single C_{60} molecules, and the red arrows marks the G_0 peak.

Incidentally, how is the y-axis in Figure 4(a-c) calibrated? The junction extensions of only ca. 0.4 nm seem to suggest that, even allowing for the usual snap-back distance, the molecules are not in fact being fully lifted in the junction (contrary to the cartoon in Fig. 2). Is it not

possible that the conductance increase is indeed due to C60 interacting in some way with the molecule in the junction (either in the cavity for 6, or more peripherally for 5), but as the molecule is lifted, the C60 adheres strongly to the gold surface and it is actually this which causes the junctions to break down?

4. Reply: In Figs.4a-c, the y axis is the conductance, and the x axis is the relative stretching distance. Figs.4a-c are generated by detecting the beginning and end of conductance plateaus and aligning the beginning of each curve at the distance origin. Those parts before the junction formation and after the junction breakdown are excluded from the 2D histogram. As described in our reply to question #1, since G0 is out of the measurable conductance range, a conventional stretching distance, measured from the point contact of electrodes, is not available. As a result, the plateau length on conductance-distance traces represents the distance of the molecular junction being stretched from formation to breakdown. Fig. 8 (added to SI as Fig. S18) shows that the plateau length is the longest for **6**, and the host-guest complexes demonstrate comparable, but slightly shorter plateau lengths than the free metallocycles. This is intuitively reasonable, because **6** is expected to have the largest flexibility when connected with electrodes, and the incorporation of C_{60} decreases the junction flexibility.

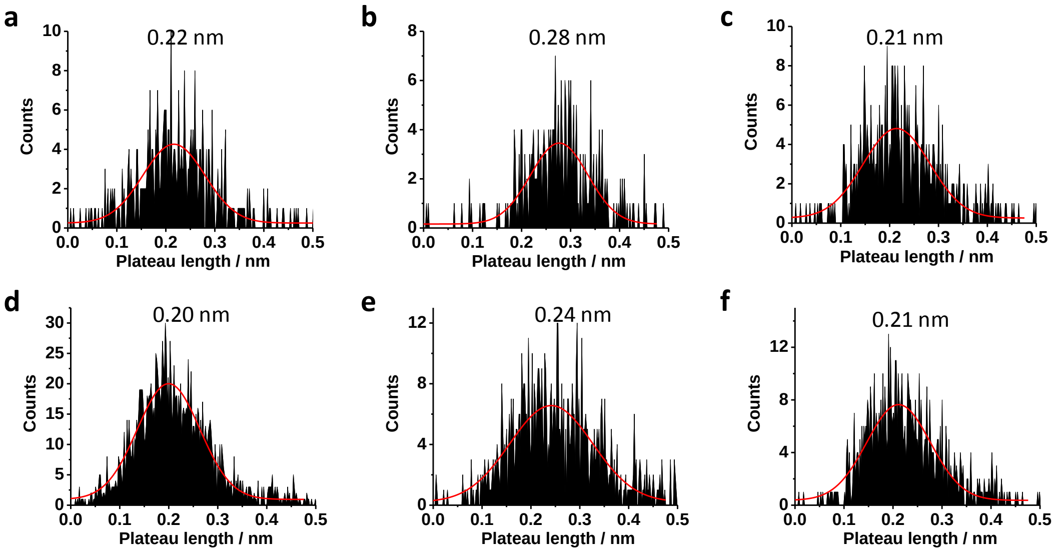

Fig. 8. Histograms of the plateau length in conductance versus distance traces of **5** (a), [**5** + C_{60}] (d), **6** (b), [**6** + C_{60}] (e), **7** (c), **7** incubated with C_{60} (f) . Red curves are Gaussian fittings of the plateau length peaks. The numbers marks the peak position of Gaussian fittings.

In the theory section of the paper, T(E) curves are used to support the notion of (i) conductance increase on complexation and (ii) conductance decrease on molecular distortion. How well (or otherwise) do the theoretical conductance values for the molecules and their complexes correlate with experiment?

5. Reply: We thank the reviewer for this point.

(i) Upon complexation with C_{60}, the theoretical conductance values show an increase in conductance compared with the bare metallocycles. This trend correlates well with our experiments and is insensitive to the binding configuration of the molecule to the electrodes. To illustrate this last point, we have provided new results in Fig. 9 and 10 below (added to the SI as Figs. S36 and S37), in which one of the pyramidal electrode tips used in Fig 4 of the manuscript is replaced by a flat electrode. Comparison between Figs. 9 and 10 below and Fig. 4 of the manuscript shows that although the above trend is resilient, the precise values of the conductances are sensitive to the shape of the electrodes. The conductances with a flat substrate and pyramidal tip are closer to the experimental values, which suggest that for our experiments a combination of a flat substrate electrode and pyramidal tip is encountered with high probability.

(ii) Regarding the conductance decrease due to distortion, as noted in the text above, and below figure 5 in main manuscript, the conductance of the distorted host **5'** is lower than that of the undistorted host **5**, whereas the conductance of the (slightly) distorted host **6'** is a little higher than that of the undistorted host **6**. Therefore, we conclude that distortion affects the conductance of the host, but the shift could be either to higher or lower conductance. The distorted geometries are shown in more detail in Fig. 11 (added to SI as Fig. S35) below. The slightly higher conductance of **6'** compared with **6** is due to a combination of a number of small geometric effects. For example, the shorter distance 1.9 nm for **6'** between the two N atoms connected to gold electrodes compared to 2.1 nm for **6** indicated by the vertical yellow lines and the smaller spacing between the -Pt-$P(CH_3)_3$ groups (indicated by the two dashed ellipses) in **6'** could account for the slight increase. The shorter distance and smaller spacing are due to the attraction of C_{60} in [**6**+C_{60}] complex before removing C_{60}. In contrast, the lower conductance of **5'** compared to **5** in Fig. 5g is due to the severe deformation of the planar geometry from the lateral view. In this case N-N distances differ significantly (they are 1.81 nm and 1.78 nm for **5** and **5'** respectively) and C_{60} cannot enter the host fully as shown by Fig. 5b of the main script. In the experiment, the distortion effect cannot be isolated from the effect of guest binding, because distortion of the host occurs at the same time as binding of the C_{60}.

To address this point, we have modified the text above Fig. 5 of the main manuscript as follows:

After removing C_{60} from the host-guest complex junctions, while freezing the metallocycle, the transmission functions of the distorted hosts **5'** and **6'** (shown by the yellow curves in Fig. 5g, h) are lower than those of [**5** + C_{60}] and [**6** + C_{60}]. This indicates the presence of an extra current path in the complexes due to the presence of C_{60}. Fig. 5g reveals that the transmission of **5'** is also significantly lower than that of the undistorted **5**, which shows that the distortion of the host (shown in Fig. 5b) can also have a significant effect on the conductance. On the other hand, Fig. 5h reveals that the transmission of **6'** is much closer to that of **6**, which shows that the smaller distortion of this host (shown in Fig. 5d) has a less significant effect on the conductance. Since the transmission of **5'** is lower than **5**, while the transmission of **6'** is slightly higher than that of **6**, we conclude that the distortion of the host can either increase or decrease the conductance and the effect of distortion is more pronounced in **5'** than in **6'**. More geometric details are shown in Fig. S35.

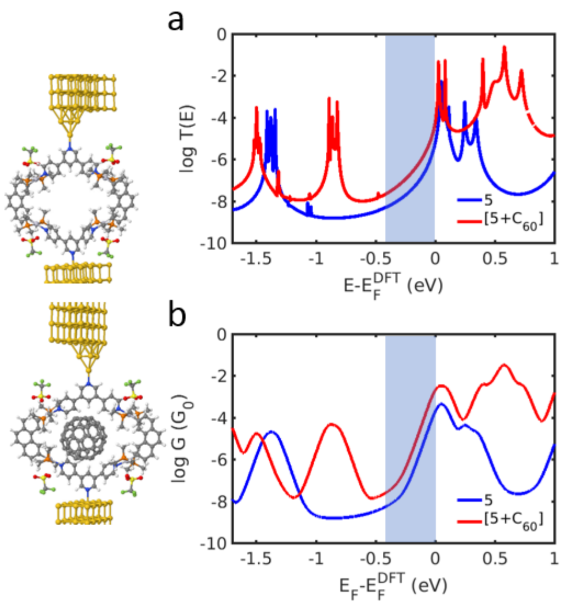

Fig. 9. Configurations for molecule **5** and complex [**5**+C_{60}] and the corresponding transmission spectra, when the lower electrode is flat and the upper electrode is pyramidal. a. Transmission functions as the function of energy. b. Conductance curves as the function of Fermi energy referring to that estimated by DFT for **5** and [**5**+C_{60}].

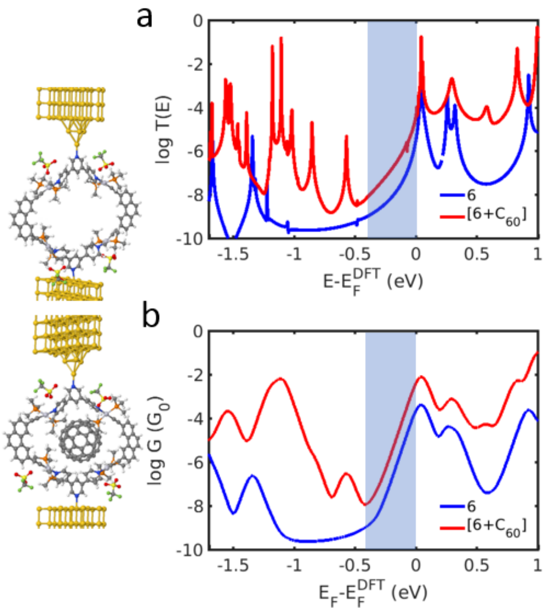

Fig. 10. Configurations for molecule **6** and complex [**6**+C_{60}] and the corresponding transmission spectra, when the lower electrode is flat and the upper electrode is pyramidal. a. Transmission functions as the function of energy. b. Conductance curves as the function of Fermi energy referring to that estimated by DFT for **6** and [**6**+C_{60}].

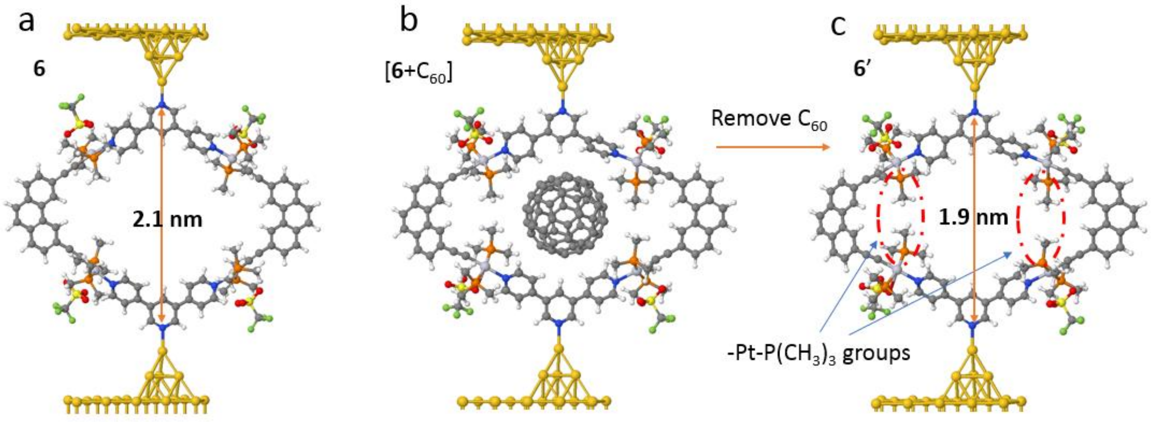

Fig. 11. Geometries of **6**, [**6**+C_{60}], **6'** which corresponds to the transmission spectra in Fig. 5h.

The complex between 6 and C60 is clearly a charge transfer interaction from the electronic spectroscopy data, so: does this result in a Fano resonance near the contact Fermi energy as seen by another of the corresponding authors Lambert (et al) in their recent paper on oligothiophene-TCNE charge transfer complexes (Nanoscale 2015, 7, 18949)? It is hard to tell from the Figures, as these are pyridine-contacted molecules and there is the usual proximity of the LUMO resonances to E(F) seen with this contact group, although there does seem to be an additional peak for the complex with 6. (Incidentally, why is the latter paper not referenced? It would seem to be relevant here).

6. Reply: We thank the reviewer for highlighting this point. In contrast with the oligothiophene-TCNE charge transfer complexes discussed in the above Nanoscale paper, charge transfer does not result in Fano resonances, because Fano resonances occur when a pendant orbital sits orthogonal to the current path and weakly couples to the current-carrying backbone. In our host-guest complexes, the bound states on the C_{60} form part of the current path and therefore lead to Breit-Wigner resonances, rather than Fano resonances. On the other hand, as discussed in the above Nanoscale paper, charge transfer can cause energy levels on the donor or acceptor to move towards the Fermi level and therefore the resonance (Fano or otherwise) tends to appear near E_F. This effect is clearly present in the red curves of Fig. 5 of the main manuscript, where the resonances associated with the C_{60} LUMO are pinned close the DFT-predicted Fermi energy.

To clarify this point, the text has been included in the discussion below Fig. 6 of the main manuscript:

In contrast with the oligothiophene-TCNE charge transfer complexes[56], the host-guest charge transfer does not result in Fano resonances, because Fano resonances occur when a pendant orbital weakly couples to a current-carrying backbone and sits orthogonal to the current path. In our host-guest complexes, the bound states on the C_{60} form part of the current path and therefore lead to Breit-Wigner resonances, rather than Fano resonances. However, as discussed in oligothiophene-TCNE complexes[56], charge transfer can cause energy levels on the donor or acceptor to move towards the Fermi level and therefore the resonance (Fano or otherwise) tends to appear near E_F. This effect is clearly present in the red curves of Fig. 5, where the resonances associated with the C_{60} LUMO are pinned close the DFT-predicted Fermi energy. In contrast, the resonances just below the shaded regions (between -1.2 and -0.8 eV for [**5** + C_{60}] and between -1.0 and -0.5 eV for [**6** + C_{60}]) are due to states located on the host. Therefore, these HOMO resonances are sensitive to the gating effect of the negatively charged C_{60} guest. Consequently, they move down in energy as the C_{60} is systematically moved away from the host.

In summary, this might be a suitable paper for resubmission to Nature Communications with (i) stronger evidence in favor of the mechanism suggested by the authors (ii) additional surface characterization of the molecular monolayers.

7. Reply: As stated in the point-by-point reply above, we have presented additional evidence in favor of the suggested mechanism. The molecular monolayers were further studied by XPS and AFM techniques.

Reviewer# 2

There has been an increasing level of interest in determining the single molecular conductance of increasingly complex molecular and biomolecular structures and this has included structures that exploit weaker non-covalent interactions, with some of these studies being referenced (references 28-32). This present work extends such investigations to an interesting and chemically rather exquisite supramolecular assembly which involves the insertion of C60. Tang et al show here that conductance of organoplatinum(II) metallocycle hosts can be significant enhanced by the incorporation of a fullerene guest molecule. This is shown using the STM- break junction method (STM-BJ), a very highly cited and respected method for molecular conductance determination which was invented by one of the co-authors (Nongjan Tao) and Bingqian Xu. Conceptually new here is the demonstration that mechanically stretching the metallocycle-C60 junction with this technique can induce the expulsion of the fullerene guest from the metallocycle. This then provides a form of mechanical conductance switch.

I support publication but would recommend some additions to underpin the STM measurements section as listed below:

(1) The authors should demonstrate that the junction breaking distances are comparable for the with and without C60 cases. I can believe that the junction is fully elongated with the C60 in place as in the cartoon in Figure 2 (top right) but this may not be the case with the empty macrocycles which contain a plethora of possible STM tip attachment points and can also tilt towards the surfaces. This can be straightforwardly resolved by presenting conventional 2D histograms of junction conductance versus stretching distance to show that the empty macrocycle is placed at its break off point as shown the cartoon in Figure 2 (top left).

1. Reply: We thank the reviewer for these constructive suggestions. As suggested, we generated the 2D conductance histograms of the free **5**, **6** and **7** (Figs. 12, as shown in SI Fig. S16) for comparison with the 2D histograms of host-guest complexes (Figs. 4a-c), and analyzed the average plateau length of **5**, [**5**+C_{60}], **6**, [**6**+C_{60}], **7**, [**7**+C_{60}] in Fig. 8 (added to SI as Fig. S18). As a result, the plateau length on conductance-distance traces represents the distance of the molecular junction when stretched from formation to breakdown. The analysis shows that the plateau lengths of the free metallocycles (**5** and **6**) are comparable to but slightly larger than those of the host-guest complexes ([**5**+C_{60}] and [**6**+C_{60}]), suggesting that at the breakdown point, the free metallocycles are placed as shown in main text Fig.2 (top left).

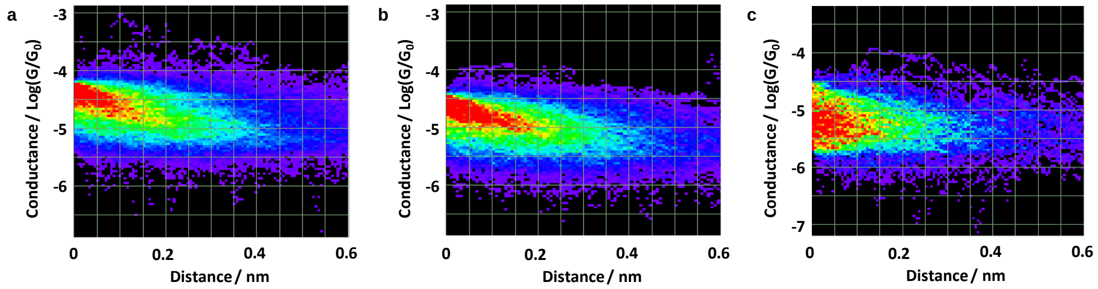

Fig. 12. 2D conductance-distance histograms of free metallocycles **5** (a), **6** (b) and **7** (c). Only the plateau region is included. The counts are represented by the color in 2D histograms.

(2) I would also suggest presenting the 1 (and 2D mentioned above) histograms to show the G(0) peaks from the break junction breaking since this reinforces the data quality.

2. Reply: As described in the response to Reviewer# 1's question, a linear voltage amplifier was used here to achieve high accuracy of conductance measurement, but its conductance range is limited to ~4 orders of magnitude and gold quantum point contact conductance (G_0) falls out of the measurable range of the liner amplifier..

(3) On page 5 can the implications of the following statement for the STM-BJ results be further clarified "The failure to observe potential CT absorption band from the complexation of 5 with C60 is possibly caused by the relatively weak and shallow interaction between them. This is supported by the DFT calculation results discussed below." If the interaction is weak and shallow why is the host-guest complexation peak seen in figure 3d as just a large peak as for 6 + C60 in Figure 3e?

3. Reply: The following discussion has been added in the revised manuscript:

Although the absorption spectral analysis implies that the interaction between **5** and C_{60} is weaker compared to that between **6** and C_{60}, the metallocycle **5** can still hold C_{60} with a shallower host-guest configuration according to our DFT calculations. This allows the complexed molecular wire, either in the case of [**5** + C_{60}] or [**6** + C_{60}], to open up the C_{60}-related current path to enhance the conductance (see transmission calculation below).

Reviewer# 3

This paper reports on the single-molecule conductance of a host-guest complex. Organoplatnium metallocycles are synthesized and their conductance measured with the STMBJ technique. The molecular conductance can be increased by one order of magnitude when a C60 molecule is encapsulated. Conductance-distance histograms for two metallocycles show two plateaus, which are interpreted as corresponding to the free host (low conductance) and host-C60 complex (high conductance). When increasing the tip distance the C60 molecule is released and the conductance of the free host is recovered. Theoretical calculations aid in the interpretation of measurements. The results are compelling and add to a growing number of papers investigating conductance of host-guest complexes. I recommend publication but some issues need to be addressed first.

The increase in conductance due to C60 seems convincing in view of the STMBJ histograms of figure 3. New conductance peaks are observed for the [5+C60] and [6+C60] complexes. The large cavity size of 7 suggests no sufficient host-guest interaction. The paper mentions mechanical control of host-guest interactions as an attractive strategy however the conductance switch described by the authors seems to take place in one direction only (release of C60). Can encapsulation be induced?

1. Reply: We thank the reviewer for highlighting this point. Due to the limit of junction stability and kinetics of the encapsulation step, statistical measurement and analysis of the re-encapsulation process is difficult. However, to elucidate the mechanical effect of conductance switching, we performed 'pull and hold'experiments, i.e., to stop the tip movement once a junction formation is detected. As shown in Fig. 6 (as shown in SI Fig. S17), the higher conductance states of [**5**+C_{60}] and [**6**+C_{60}] persist for more than 0.1 s and 0.2 s, respectively. This is in contrast with the conventional break junction measurements (19.5 nm/s retraction rate) where the high conductance states persist for less than 0.03 s (0.59 nm at 19.5 nm/s retraction rate) in any case. The above results confirm the assumption that the conductance switching from higher to lower level is mechanically dependent.

Control experiments with C60 solution showed no conductance peak, contrary to reports in the literature for this molecule, why?

2. Reply: The concentration of C_{60} in our experiments was low (10 M). After rinsing the substrate with copious amounts of solvent, the coverage of C_{60} was likely too low (ref as in 45-50). To verify this, we modified the Au surface using a higher concentration of C_{60} solution (7 mM) and removed the excessive C_{60} by transferring the surface to 1,1,2,2-tetrachloroethane shortly (ref 48). Fig. 7 (added to SI as Fig. S19) shows the conductance histogram of C_{60} modified on Au surface, featured by two conductance peaks at around 0.2 G_0 and 0.5 G_0, which agrees with the reported values (ref 45, 49). As the reply of question 3.2 of Reviewer#1.

Theoretical calculations demonstrate the potential to modulate host-guest interactions by changing host size. Transmission calculations show that C60 increases the conductance of the host providing an extra current pathway. Theoretical conductance is smaller than experimental values, in contradiction to the results of DFT calculations in the literature. This should have been discussed but was not mentioned. The enhancement of conductance by the C60 host is expected and is demonstrated by the calculations. However, there seems to be a fundamental disagreement between calculations and experiment where calculations show less conductance, not more.

3. Reply: We thank the review for this point. Whereas the increase in conductance due to host-guest binding is independent of the shape of the electrodes, the predicted conductance values are not. Due to the large-scale nature of these simulations, it is only possible to sample a small number of tip configurations theoretically. Nevertheless, to address this point, we have carried out new simulations, in which one of the pyramidal electrode tips (used to obtain the results in Fig. 4 of the manuscript) is replaced by a flat electrode. Figs 9 and 10 show the new transmission curves and predicted conductances. Comparison between these results and Fig. 4 of the manuscript shows that although the conductance increase upon guest binding is resilient, the precise values of the conductances are sensitive to the shapes of the electrodes. The conductances with a flat substrate and pyramidal tip are closer to the experimental values. Therefore, we conclude that for our experiments a combination of a flat substrate electrode and pyramidal tip is encountered with high probability.

To address this point, the following text has been added to the manuscript in the paragraph preceding the conclusion and the new figures included in the SI as Figs. S36 and S37).

Whereas the increase in conductance due to host-guest binding is independent of the shape of the electrodes, the predicted conductance values are not. Due to the large-scale nature of these simulations, it is only possible to sample a small number of tip configurations theoretically. Nevertheless, to illustrate this point, we have carried out new simulations, in which one of the pyramidal electrode tips (used to obtain the results in Fig. 5) is replaced by a flat electrode. Figs. S36 and S37 show the new transmission curves and predicted conductances.

Comparison between these new results and Fig. 5 shows that although the conductance increase upon guest binding is resilient, the precise values of the conductances are sensitive to the shape of the electrode. The conductances with a flat substrate and pyramidal tip are closer to the experimental values. Therefore, we conclude that for our experiments a combination of a flat substrate electrode and pyramidal tip is a closer representation of the experimental setup.

The effect of donor-acceptor interactions in the metallocycle-C60 calculations needs some clarification. Host-guest charge transfer shifts the frontier orbitals of C60 (eg. through the intermediate configurations in figure 6). The shift in C60 HOMO is very large however the LUMO is unchanged. The LUMO of the C60 acceptor in the host-guest is not filled, why not?

4. Reply: We thank the reviewer for highlighting this point. As discussed in reply 6 to reviewer#1, in common with other charge transfer complexes, such as those discussed in [Nanoscale 2015 7, 18949], charge transfer causes energy levels of the donor or acceptor to move towards the Fermi level and therefore a resonance tends to appear near E_F. This effect is clearly present in the red curves of Fig. 5 of the main manuscript, where the resonances associated with the C_{60} LUMO appear close the DFT-predicted Fermi energy, just above the shaded regions. The LUMO of the C_{60} (rather than the HOMO) moves towards E_F, because the C_{60} accepts a fraction of an electron from the host and becomes partially filled. Consequently the Fermi energy is located in the low energy tail of the lifetime-broadened LUMO. As an example from the literature (Phys. Rev. B, 63, 121104), studied a C_{60} in direct contact with Au electrodes and showed that electrons were transferred into the triply degenerate LUMO of the C_{60}. In other words, the LUMO is partly filled. In our case the C_{60} is not in direct contact with the gold and we find that 0.62 (0.48) of an electron is transferred from the gold into the triply-degenerate LUMO of the C_{60} when located in host **6** (**5**) respectively.

This pinning of the C_{60} LUMO to the vicinity of the Fermi energy is why the LUMO does not shift through the intermediate configurations in Fig. 6. On the other hand, as discussed in the main text below Fig. 6, the resonances just below the shaded regions (between -1.2 and -0.8 eV for **5**+C_{60} and between -1.0 and -0.5 eV for **6**+C_{60}) are due to states located on the host. Therefore these HOMO resonances are sensitive to the gating effect of the negatively charged C_{60} guest, which increases their energy through electrostatic repulsion. Consequently they move down in energy as the C_{60} is systematically moved away from the host.

This point is addressed by the following modified text in the main manuscript below Fig. 6.

….. as discussed in oligothiophene-TCNE complexes[56], charge transfer can cause energy levels on the donor or acceptor to move towards the Fermi level and therefore the resonance (Fano or otherwise) tends to appear near E_F. This effect is clearly present in the red curves of Fig. 5, where the resonances associated with the C_{60} LUMO are pinned close the DFT-predicted Fermi energy. In contrast, the resonances just below the shaded regions (between -1.2 and -0.8 eV for [**5** + C_{60}] and between -1.0 and -0.5 eV for [**6** + C_{60}]) are due to states located on the host. Therefore, these HOMO resonances are sensitive to the gating effect of the negatively charged C_{60} guest. Consequently, they move down in energy as the C_{60} is systematically moved away from the host.

A 100 times increase in transmission was obtained when the Au-N bond was almost perpendicular to the plane of the pyridine ring. Although intriguing, without any additional information it is not possible to judge whether this geometry is in any way relevant or energetically favorable. Is such a change in conductance observed in the STMBJ experiments?

5. Reply: Thanks very much for raising this point. The “around a 100 times increase” mentioned in the paragraph before the conclusion is a typo and should be replaced by “around a 10 times increase.”

The geometry shown in Fig. S34, in which the Au-N bonds is almost perpendicular to the plane, is one of many that could be encountered during the evolution of a junction pulling curve. Since the electrodes are closer together in Fig S34, compared with the electrodes in Fig. 4 of the main text, we expect the former to be encountered before the latter during junction pulling. As the junction evolves from the former to the latter, we therefore expect the conductance to drop by a factor of approximately 10. Decreases of this order of magnitude are indeed present in the pulling curves of Fig. 2 of the main script.

To address this point, the typo has been corrected and the below text has been added to the paragraph above the conclusion.

…..Since the electrodes are closer together in Fig S34, compared with the electrodes in Fig. 5, we expect the former to be encountered before the latter during junction pulling. As the junction evolves from the former to the latter, we therefore expect the conductance to drop by a factor of approximately 10. Decreases of this order of magnitude are indeed present in the pulling curves of Fig. 2.

Further analysis and discussion are necessary.

6. Reply: Further analysis and discussion are presented as above point-by-point reply.

Reviewers' Comments:

Reviewer #1:
Remarks to the Author:
The authors have addressed all of the comments and suggestions of the referees in a satisfactory way. In particular, the additional characterization data is useful and convincing. Although the question of why molecule 5 does not significantly complex C60 in solution, but does appear to do so in the junctions, is still an open one, the paper should now be published in its revised form. One caveat; the additional text in the revised version contains some grammatical errors and could benefit from a careful re-reading by the authors and/or editorial attention before final publication.

Reviewer #2:
Remarks to the Author:
I did not find the additional experiments added after review to be sufficiently convincing. In terms of the XPS the atomic percentages could approximately remain constant even after partial fragmentation on the surface. The AFM imaging is insufficient and would use best flat single crystal gold as a starting place rather than rough. The pull and hold experiments on junction stability are a nice addition but not decisive. Proper 2D histogram measured from a point contact of electrodes are not available and the 2D stretching ones of Figure 4 are not a substitute in terms of assessing whether the molecule is properly pulled in the junction. These data need strengthening before publication can be recommended.

Reviewer #3:
Remarks to the Author:
In this version some of the issues raised have been answered however other questions related to the calculations are not addressed.

While calculations taken on their own establish that complexation with C60 increases conductance, I am not convinced they represent the experimental situation.

The authors propose a combination of flat substrate electrode and pyramidal tip because they are closer to the experimental values. However it has been argued that pyridine groups bind preferentially to adatoms and not to flat substrates (Nature Nanotechnology volume 4, pages 230–234 (2009)). Here there are no binding energies presented to show that the proposed binding is realistic. To make matters more confusing, the data of gold-molecule-gold calculations in the paper still have two pyramidal electrode tips despite the combination of flat substrate electrode and pyramidal tip favored by the authors. Similar analysis is missing also for the geometry in which the Au-N bond is almost perpendicular to the plane, what is the binding energy? There are no arguments supporting the claim that the adsorption geometry on a flat surface, or other geometries, are stable or favorable.

The paper does not really discuss how well the theoretical conductance correlates with experiment. In their reply, the authors just state that the precise values of the conductances are sensitive to the shape of the electrode, which is hardly a new result. The suggested geometries seem to be suggested solely to achieve a better agreement with experiment with no further analysis.

I am not convinced the theoretical calculations represent the geometries encountered in the experiments and I therefore cannot recommend publication.

Reviewers' comments:

Reviewer #1:

The authors have addressed all of the comments and suggestions of the referees in a satisfactory way. In particular, the additional characterization data is useful and convincing. Although the question of why molecule 5 does not significantly complex C60 in solution, but does appear to do so in the junctions, is still an open one, the paper should now be published in its revised form. One caveat; the additional text in the revised version contains some grammatical errors and could benefit from a careful re-reading by the authors and/or editorial attention before final publication.

Reply: We gratefully thank Reviewer #1 for his/her recommendation for the publication of our work. we have checked and corrected the grammatical errors in the manuscript.

Reviewer #2:

I did not find the additional experiments added after review to be sufficiently convincing. In terms of the XPS the atomic percentages could approximately remain constant even after partial fragmentation on the surface. The AFM imaging is insufficient and would use best flat single crystal gold as a starting place rather than rough. The pull and hold experiments on junction stability are a nice addition but not decisive. Proper 2D histogram measured from a point contact of electrodes are not available and the 2D stretching ones of Figure 4 are not a substitute in terms of assessing whether the molecule is properly pulled in the junction. These data need strengthening before publication can be recommended.

Comments from reviewer#2 last review: "The authors should demonstrate that the junction breaking distances are comparable for the with and without C60 cases. I can believe that the junction is fully elongated with the C60 in place as in the cartoon in Figure 2 (top right) but this may not be the case with the empty macrocycles which contain a plethora of possible STM tip attachment points and can also tilt towards the surfaces. This can be straightforwardly resolved by presenting conventional 2D histograms of junction conductance versus stretching distance to show that the empty macrocycle is placed at its break off point as shown the cartoon in Figure 2 (top left)."

Reply: We thank the reviewer for suggesting the additional control experiment. We have now completed the experiment and determined 1D and 2D histograms with conductance ranging from electrode quantum point contact to the noise level with a logarithmic current-voltage converter. These histograms (for **5**, **6**, [**5** + C_{60}] and [**6** + C_{60}]) are shown in Fig. 1, and Fig. S21 of SI. As summarized in Table 1 (Table S1 of SI), the conductance values measured by logarithmic amplifier and linear amplifier are in agreement within 13.5% (which is within the expected difference between logarithmic and linear amplifiers).

The relative displacement distributions from electrodes' point of contact to junction break are shown in Fig. 2 (Fig. S22 of SI). After adding the Au-Au snap-back distance (0.5 nm), the junction breaking distances of **5**, [**5** + C_{60}], **6** and [**6** + C_{60}] are 1.38 nm, 1.38 nm, 1.41 nm, and 1.42 nm, respectively, suggesting similar junction breakdown configurations for the metallocycles before and after encapsulation of C_{60}. From the DFT optimized molecular length of **5** (1.79 nm) and **6** (1.94 nm), the angle between the center axis of the metallocycle and the substrate plane is estimated to be ~50° for both metallocycles at the point of junction breakdown. These results indicate that during the break junction measurements the metallocyclic molecules were lifted and distorted by the retreating STM tip. We have included these to the discussion of the revised manuscript (page 4-6).

We also thank the reviewer's suggestion about characterizing the monolayers on a flat single crystal gold substrate. We have re-measured AFM images of **5** and **6** on Au(111) surfaces. As shown in Fig. 3 (Fig. S30 of SI), we were unable to clearly resolve the metallocycles due to the limited resolution of AFM and imperfect Au(111) surface. However, the heights of the monolayers on Au(111) were determined with AFM, which are 1.2 nm and 1.3 nm for **5** and **6**, respectively, close to the STMBJ results. Regarding the stability of metallocycles on Au surfaces, we have shown previously that similar coordination assembly structures are kept intact after adsorption on Au(111) surfaces. For example, as shown in the ref 55-56, Pt(II)-based self-assembled supramolecular rectangle, square, three-dimensional cage and prism have been obtained by STM.

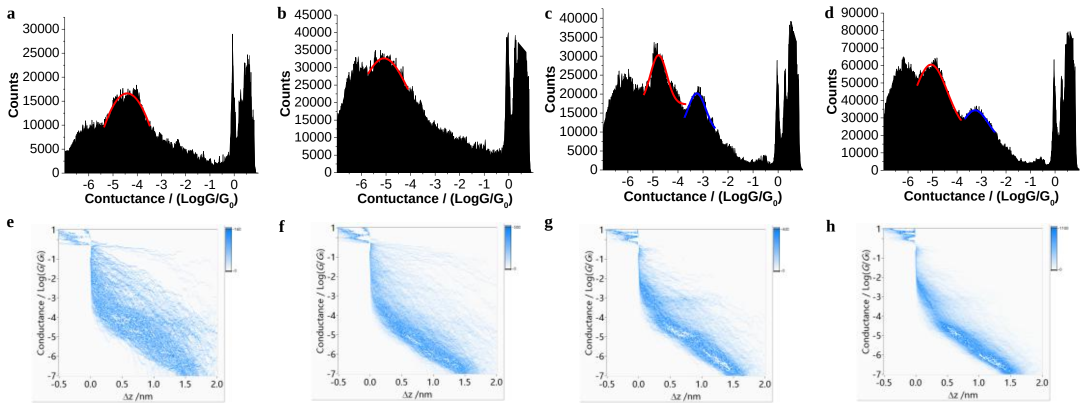

Fig. 1. 1D and 2D conductance histograms of metallocycles and their host-guest complexes with C_{60}, measured with logarithm amplifier controlled STM. a, e for **5**, b, f for **6**, c, g for [**5** + C_{60}], d, h for [**6** + C_{60}]. The red and blue curves are Gaussian fits of the conductance peaks.

Table 1. The conductance measured by logarithm and linear amplifiers.

Complex	Conductance/ Log(G/G_0) (Linear)	Conductance/ Log(G/G_0) (Log)	δ
**5**	-4.51	-4.42	2.00%
[**5**+C_{60}]	-4.47	-4.77	6.71%
	-3.55	-3.25	8.45%
**6**	-4.85	-5.08	4.74%
[**6**+C_{60}]	-5.05	-5.06	0.198%
	-3.77	-3.26	13.5%

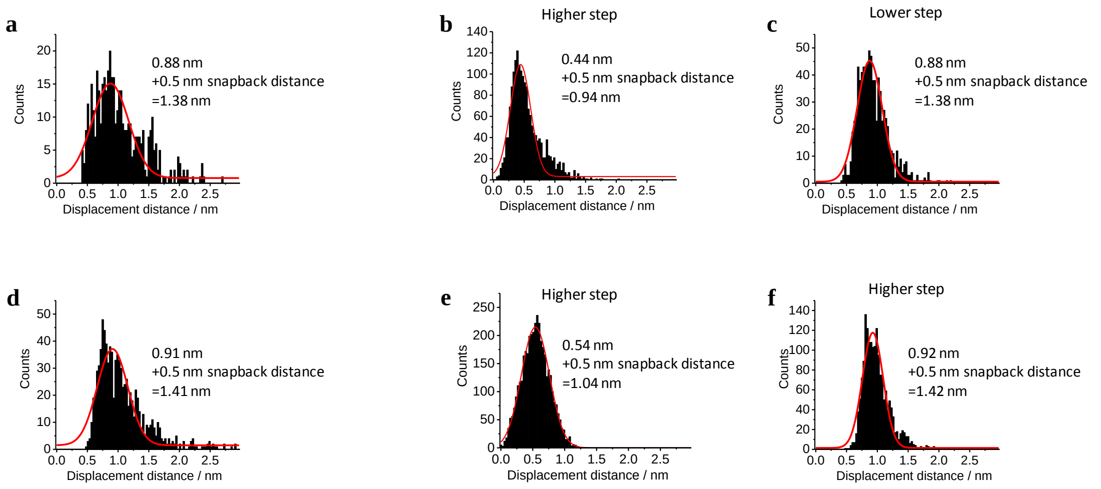

Fig. 2. The relative displacement distributions from electrodes' point of contact to . (a) junction break of **5**, (b) the sharp drop of higher plateau for [**5** + C_{60}], (c) junction break of [**5**+C_{60}], (d) junction break of **6**, (e) the sharp drop of higher plateau for [**6**+C_{60}], (f) junction break of [**6** + C_{60}]. The red curves are Gaussian fits.

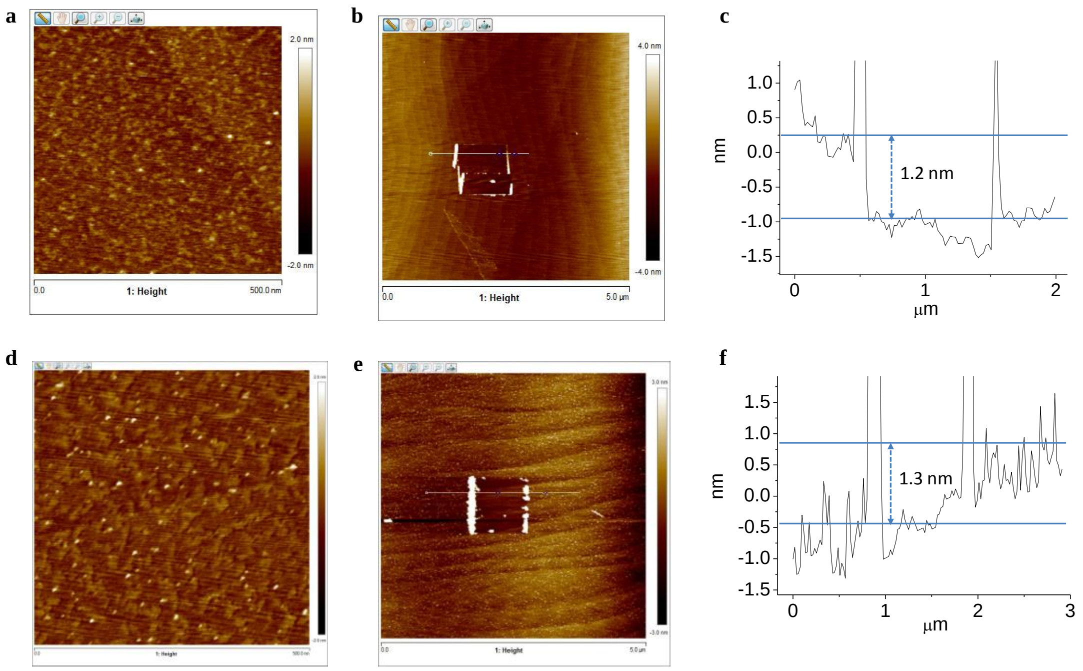

Fig. 3. AFM images in both tapping and contact modes and corresponding height changes of Au(111)/**5** (a, b, c), Au(111)/**6** (d, e, f). AFM images of (b, e) were measured after the scratching of the modified surface by the contact mode.

Reviewer #3:

In this version some of the issues raised have been answered however other questions related to the calculations are not addressed.

While calculations taken on their own establish that complexation with C60 increases conductance, I am not convinced they represent the experimental situation.

The authors propose a combination of flat substrate electrode and pyramidal tip because they are closer to the experimental values. However it has been argued that pyridine groups bind preferentially to adatoms and not to flat substrates (Nature Nanotechnology volume 4, pages 230–234 (2009)). Here there are no binding energies presented to show that the proposed binding is realistic. To make matters more confusing, the data of gold-molecule-gold calculations in the paper still have two pyramidal electrode tips despite the combination of flat substrate electrode and pyramidal tip favored by the authors. Similar analysis is missing also for the geometry in which the Au-N bond is almost perpendicular to the plane, what is the binding energy? There are no arguments supporting the claim that the adsorption geometry on a flat surface, or other geometries, are stable or favorable.

The paper does not really discuss how well the theoretical conductance correlates with experiment. In their reply, the authors just state that the precise values of the conductances are sensitive to the shape of the electrode, which is hardly a new result. The suggested geometries seem to be suggested solely to achieve a better agreement with experiment with no further analysis.

I am not convinced the theoretical calculations represent the geometries encountered in the experiments and I therefore cannot recommend publication.

Reply to reviewer #3:

The article (Nat. Nanotech. 2009, 4, 230–234) considers a 4-4’ bipyridene molecule attached to gold electrodes terminated by an adatom, dimer, trimer, pentamer or a pyramid. It demonstrates that the binding energy depends on the angle between the bipyridine long axis and the bond between the pyridine nitrogens and under-coordinated gold atoms on the tip of the electrodes. Tilting the nitrogen–gold bond out of the pyridine plane results in a decreased junction binding energy, from 1.36 eV for a vertical junction to 0.70 eV when tilted to 30 degrees. We have presented detailed results ourselves of the nature of binding between pyridine-terminated molecules and

gold electrodes (J. Am. Chem. Soc. 2012, 134, 2292−2304). On the other hand, these studies ignore important dispersion forces. When these are included (see J. Phys. Chem. C, 2013, 117, 4470) the binding energy to a flat gold (111) surface increases substantially to around 0.78 eV, which is higher than the binding energy claimed for the extended junctions in Nat. Nanotech. 2009, 4, 230–234. We entirely agree with the results of these studies and are delighted to refer to them.

To further address this point, we have calculated the binding energies to each electrode of the two geometries below, using the LDA(CA) exchange-correlation functional and counterpoise method (Mol. Phys. 1970, 19, 553–566) which eliminates basis set superposition errors (BSSE). Binding energies are calculated using the formula $E_b = E_{AB} - E_{a,B} - E_{A,b}$. All binding energies are significantly greater than $k_B T$ at room temperature, and therefore the junctions are stable (Fig.4, Fig. S41 of SI).

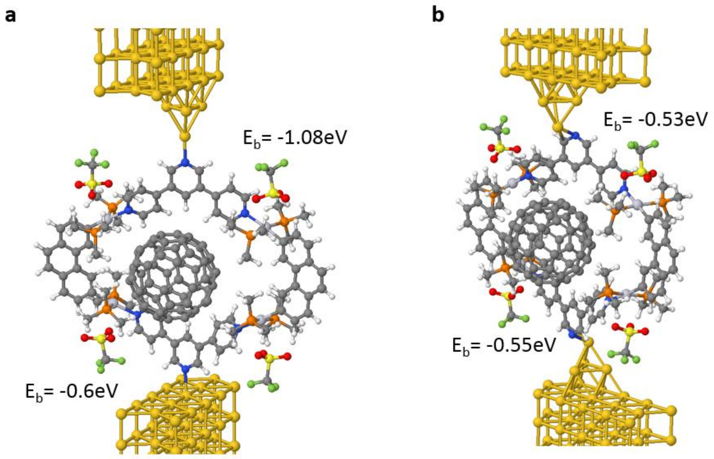

Fig. 4. Binding energies for tip-flat geometry (a) and tip-tip tilted geometry (b).

However our paper is not aimed at repeating literature studies of binding energies between pyridines and various tip geometries. Indeed the inclusion of dispersion forces in the simulation of large metallocycle-C_{60} complexes is impossible with currently available computational resources. Instead, our aim is to study the supramolecular binding of C_{60} to a metallocycle. As examples, we have presented results for both pyramidal (main article Fig. 5 and SI Fig. S37) and flat electrodes (SI Figs. S39-S40) and find that in both cases, the predicted host-guest interactions are consistent with our experiments.

To address this issue, we have included the following section in the SI:

Binding energies to gold electrodes.

The article [1] considers a 4-4' bipyridene molecule attached to gold electrodes terminated by an adatom, dimer, trimer, pentamer or a pyramid. It demonstrates that the binding energy depends on the angle between the bipyridine long axis and the bond between the pyridine nitrogens and under-coordinated gold atoms on the tip of the electrodes. Tilting the nitrogen–gold bond out of the pyridine plane results in a decreased junction binding energy, from 1.36 eV for a vertical junction to 0.70 eV when tilted to 30 degrees. The binding energy of pyridyl anchors to gold electrodes was also studied in article [2]. On the other hand, these studies ignore important dispersion forces. When these are included the binding energy to a flat gold (111) surface increases substantially to around 0.78 eV [3]. As examples of these possibilities, we have presented results for both pyramidal (Fig. S33 and Fig. S37 of SI) and flat electrodes (Fig. S39, S40 of SI) and find that in both cases, the predicted host-guest interactions are consistent with our experiments. We have also calculated the binding energies to each electrode of the two geometries below, using the LDA(CA) exchange-correlation functional and counterpoise method [4] which eliminates basis set superposition errors (BSSE). Binding energies are calculated using the formula $E_b = E_{AB} - E_{a,B} - E_{A,b}$. Even without the inclusion of dispersive forces, all binding energies are significantly greater than $k_B T$ at room temperature and therefore the junctions shown in Figs. S37, S39, S40 are stable.

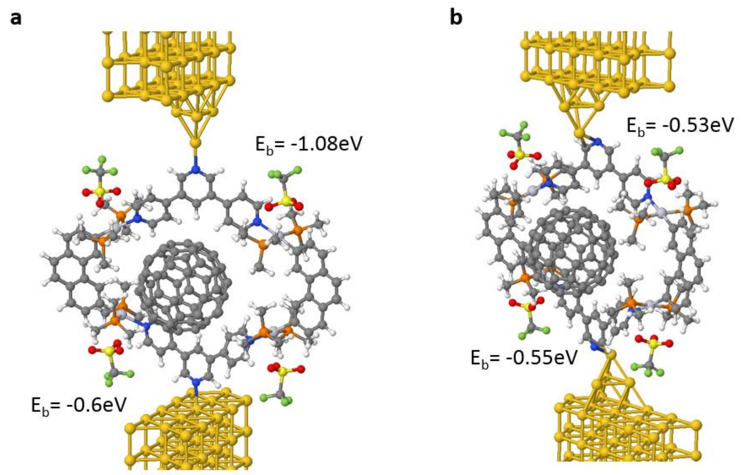

Fig. S41. Binding energies E_b to each electrode for tip-flat geometry (a) and tip-tip tilted geometry (b).

[1] Su Ying Quek, Maria Kamenetska, Michael L. Steigerwald, Hyoung Joon Choi, Steven G. Louie, Mark S. Hybertsen, J. B. Neaton and Latha Venkataraman, Nat. Nanotech. 2009, 4, 230–234.

[2] Wenjing Hong, David Zsolt Manrique, Pavel Moreno-García, Murat Gulcur, Artem Mishchenko, Colin J. Lambert, Martin R. Bryce, and Thomas Wandlowski, J. Am. Chem. Soc. 2012, 134, 2292−2304.

[3] D. Mollenhauer, N. Gasto, E. Voloshina and B. Paulus, J. Phys. Chem. C 2013, 117, 4470-4479.

[4] S. F. Boys and F. Bernardi, Mol. Phys. 1970, 19, 553–566.

And the following sentences have been added in main manuscript.

The binding energy calculation shows -1.08 eV for pyramidal tip Au-N, -0.6 eV for atop Au-N with flat gold electrode, -0.53 eV for Au-N bond out of pyridine plane, which are significantly greater than k_BT at room temperature and therefore the junctions shown in Fig. S37, S39, S40 are stable (see details in Fig. S41 of SI).

As described in the reply to referee 2, to obtain the information about the actual geometries encountered in the experiments, one of the authors went to Prof. Wenjing Hong's lab (Xiamen University) to measure the 2D histograms from electrodes' point of contact using their logarithm amplifier controlled STM. The resulting 1D and 2D histograms are shown in Fig. S21. After adding the Au-Au snap-back distance (0.5 nm), the junction breaking distance of **5**, **6**, [**5** + C_{60}] and [**6** + C_{60}] junctions are 1.38 nm, 1.38, nm, 1.41 nm, and 1.42 nm, suggesting similar junction break down configurations for the metallocycles before and after encapsulation of C_{60}. These measurements reveal thatthe pulling distances for the high conductance plateaus of [**5** + C_{60}] and [**6** + C_{60}] are around 0.94 nm, and 1.04 nm. From the optimized molecular length of **5** (1.79 nm) and **6** (1.94 nm), the angles between the center axis of the metallocycle and the substrate plane are estimated to be around 50 degrees for both metallocycles at junction breakage, and around 30 degrees for junctions corresponding to high conductance plateaus. This is different from our previous assumption that molecules are perpendicular to the surface. Therefore, we amended

our theoretical model according to the actual junction break distance measured in the experiment as follows. Due to the existence of constraints, the N-Au bond on the electrode tip is out of the pyridine plane. As shown in Figs. 5a and 5e (below), structure with 30 degrees between a N-Au bond and a pi-orbital (denoted by the two cross red dashed lines) or 120 degrees between a N-Au bond and the pyridine plane are constructed for **[5** + C_{60}] and [**6** + C_{60}] molecules. After geometrical optimization, the angles increase to 40°, 53°, 49°, 42°. Similarly, the structure with 50 degrees between N-Au bond and pi-orbital (denoted by the two cross red dashed lines) or 120 degrees between a N-Au bond and the pyridine plane are constructed for **5** and **6** molecules or **[5** + C_{60}] after C_{60} falls out and [**6** + C_{60}] after C_{60} falls out. The corresponding transport properties are shown in Fig. 6 (below). Red curves show the transmission functions of complexes, the blue depicts that of each optimal metallocycle and the yellow shows that of the distorted metallocycle obtained from optimal complexes by removing C_{60}. This shows that the transmission function within the gap shifts to higher values compared to those of the fully extended junctions shown in Figure S33. The conductances reach to $\sim 10^{-4}$ and $\sim 10^{-6} - \sim 10^{-5}$ respectively in a large range of Fermi energies (denoted by blue shaded regions) in Figure 6b, which are in great agreement with experimental data. The red curve is higher than the yellow one which reveals the additional current path due to the complexation of C_{60} with the metallocycles. For molecule **6**, the yellow curve is higher than the bl ue curve, which is mainly due to the smaller angle between the N-Au bond and pi orbitals, while for molecule **5**, the yellow curve is close to the blue one, which is due to the large geometrical deformation caused by the complexation of C_{60} and the different contact angles during the pulling process. With the above structures, all the trends of the measured transport properties are obtained and the values of the predicted conductances are improved compared with the previous estimates obtained for fully extended junctions.

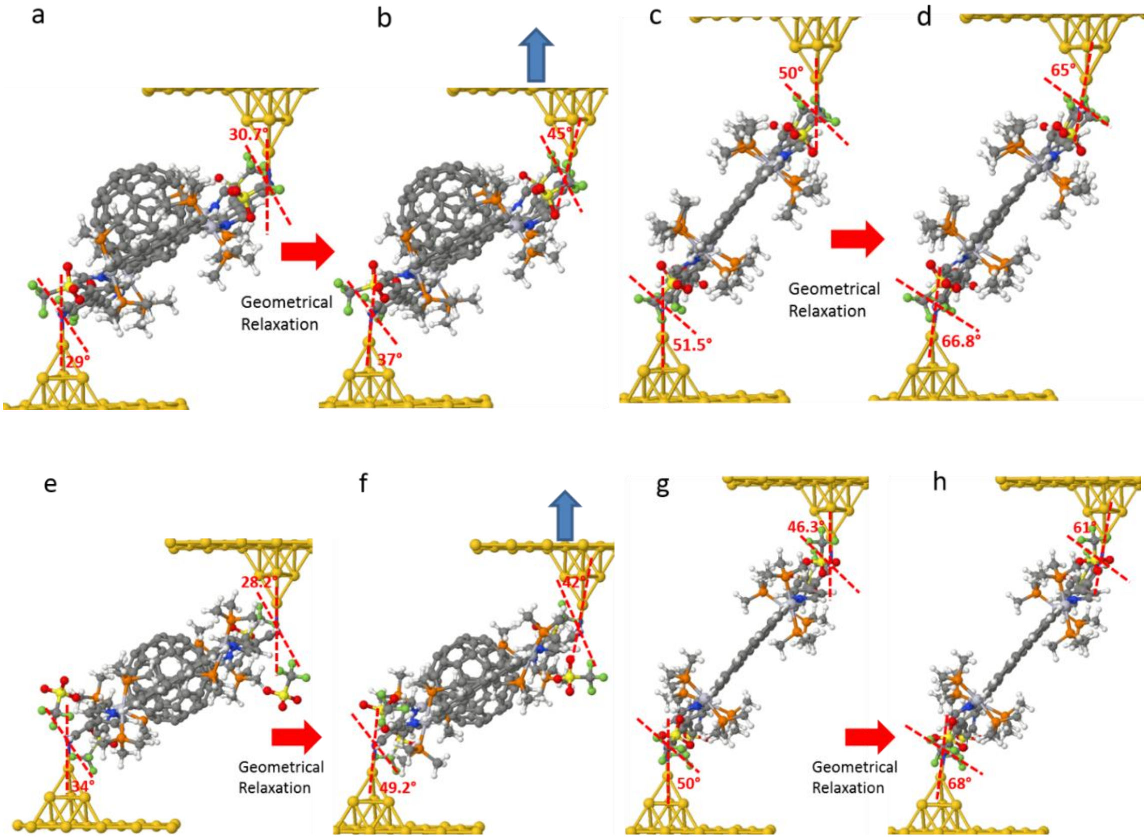

Fig. 5. Schematics of gold-molecule-gold break junctions during a pulling process. a. Initial structure corresponding to high conductance of **[5** + C_{60}]. b. Optimal structure after geometrical relaxation. c. Initial structure of 5 or **[5** + C_{60}] after C_{60} falls out. d. Optimal structure after geometrical relaxation for structure c. e. Initial structure corresponding to high conductance of **[6** + C_{60}]. f. Optimal structure after geometrical relaxation. g. Initial structure of **6** or **[6** + C_{60}] after C_{60} falls out. h. Optimal structure after geometrical relaxation for structure g.

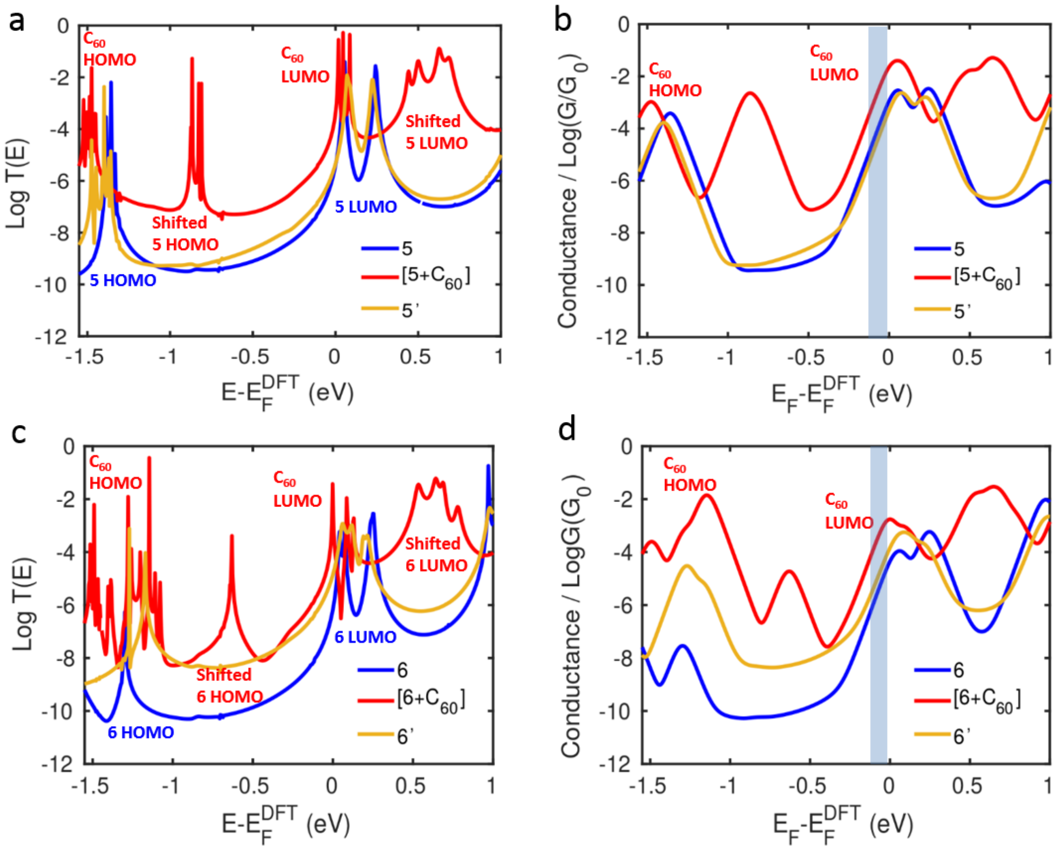

Fig. 6. Transport properties corresponding to the optimal geometries in Figure 2. a. Transmission function of molecule **5** and **[5** + C_{60}] in junction versus energy referred to Fermi energy estimated by DFT. b. Corresponding conductance evolution as the function of fermi level relative to that given by DFT. c. Transmission function of molecule **6** and **[6** + C_{60}] versus energy referred to Fermi energy estimated by DFT. d. Corresponding conductance evolution as the function of fermi level relative to that given by DFT.

The corresponding local densities of states (LDOS) and charge transfers were also calculated for the tilted-molecule junctions and as shown below, possess the same features with previous calculations for the ideal fully extended junctions.

energy window	-1.6~ -1.3eV		0 ~ 0.4eV	
**5**	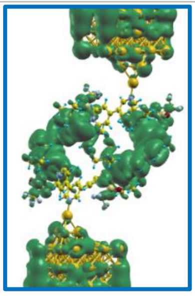		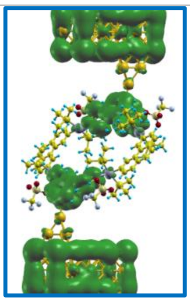	
[**5**+C_{60}]	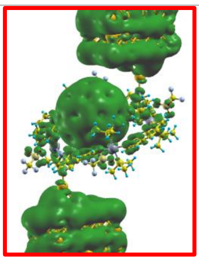	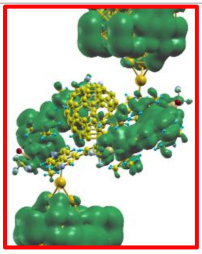	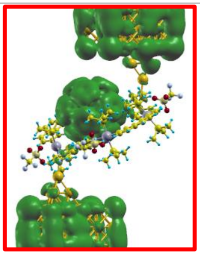	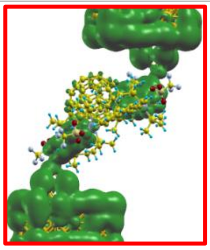
energy window	-1.6 ~ -1.4eV C_{60} HOMO	-1. ~ -0.7eV Shifted **5** HOMO	0 ~ 0.3eV C_{60} LUMO	0.3 ~1.0eV Shifted **5** LUMO

Fig. 7. Local Density of States (LDOS) of the junctions within different energy windows for tilted **5** and [**5** + C_{60}] in gold/molecule/gold junctions (Fig. S42d, b).

Energy window	-1.4 ~ -1.0 eV		0 ~ 0.5eV	
**6**	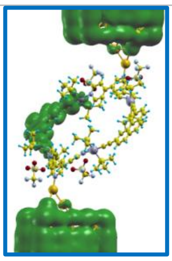		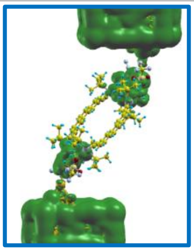	
[**6**+C_{60}]	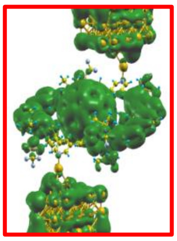	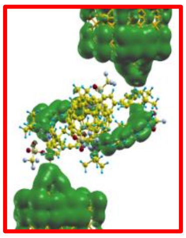	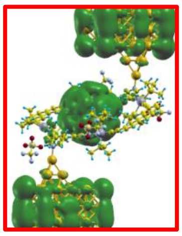	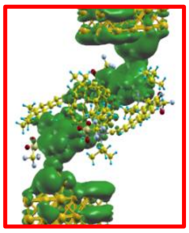
energy window	-1.6 ~ -1.3eV C_{60} HOMO	-0.8 ~ -0.5eV Shifted **6**	0 ~ 0.2eV C_{60} LUMO	0.5 ~ 0.8 eV Shifted **6** LUMO

		HOMO		

Fig. 8. Local Density of States (LDOS) of the junctions within different energy windows for tilted **6** and [**6** + C_{60}] in gold/molecule/gold junctions (Fig. S42h, f).

Table 2. Net charges of isolated complexes **5**, [**5** + C_{60}], **6**, [**6** + C_{60}] and corresponding tilted junctions (Fig. S42d, b and h, f).

	**ISOLATED COMPLEXES**		**JUNCTIONS**	
**5**	0		-0.2	
[**5**+C_{60}]	0		-0.74	
	**5**: -0.23	C_{60}: 0.23	**5**: -0.18	C_{60}: -0.56
**6**	0		-0.16	
[**6**+C_{60}]	0		-0.80	
	**6**: -0.16	C_{60}: 0.16	**6**: -0.17	C_{60}: -0.63

To address this issue, the following figure is included in the main manuscript and some paragraphs (highlighted in yellow) in the main manuscript are also modified accordingly.

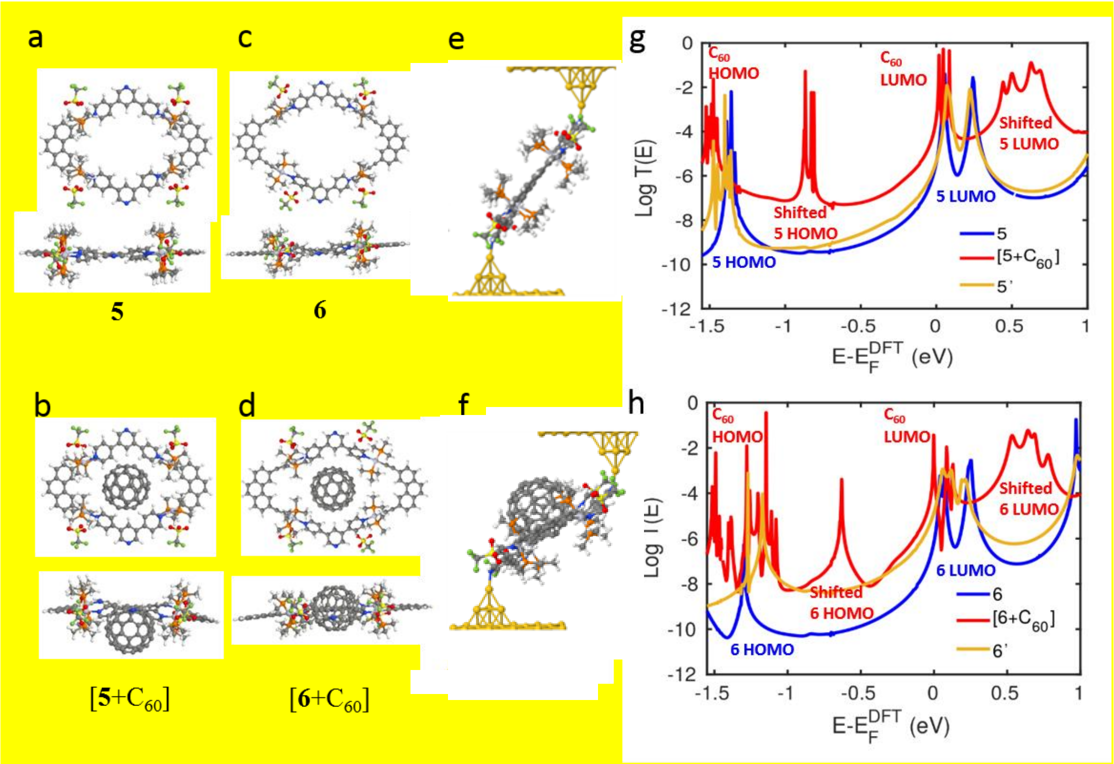

Fig. 5. Gold/molecule/gold junctions quantum calculations. Top and lateral views of metallocycle cation with -OTF anions ($CF_3SO_3^-$). **a** for **5**, **b** for [**5**+C_{60}], **c** for **6**, **d** for [**6**+C_{60}]. Single-molecule junctions containing $CF_3SO_3^-$ anions, where the terminal atom of the gold tip is bonded above and below to the nitrogen atom of the pyridine with a Au-N distance of 2.3 Å. **e** for **5**, **f** for [**5**+C_{60}] where the angles between the Au-N bond formed by electrode-apex Au and N of the connected anchor pyridine and pi-orbital of this pyridine are around 45° and 65° respectively after geometrical optimization. Quite similar junction geometries for **6** and [**6**+C_{60}] are not shown which could be found in Fig. S42 of SI. Gold atoms in the electrodes are colored yellow. In the molecule: grey, blue, white represent carbon, nitrogen, and hydrogen atoms; green, light yellow, red, large grep, orange represent fluorine, sulphur, oxygen, platinum, phosphorus atoms. **g**, Transmission functions of **5**, [**5**+C_{60}] and the distorted **5** (denoted as **5'**) which is obtained from the junction of [**5**+C_{60}] by removing C_{60}. **h**, Transmission functions of **6**, [**6**+C_{60}] and the distorted **6** (denoted as **6'**) which is obtained from the junction of [**6**+C_{60}] by removing C_{60}. The resonances of red curve are labeled by red words while the blue curve correspond to blue words. Corresponding room-temperature electric conductances are shown in Fig. S42i and S42.

In addition, the following figures have been added to the Supplementary Information.

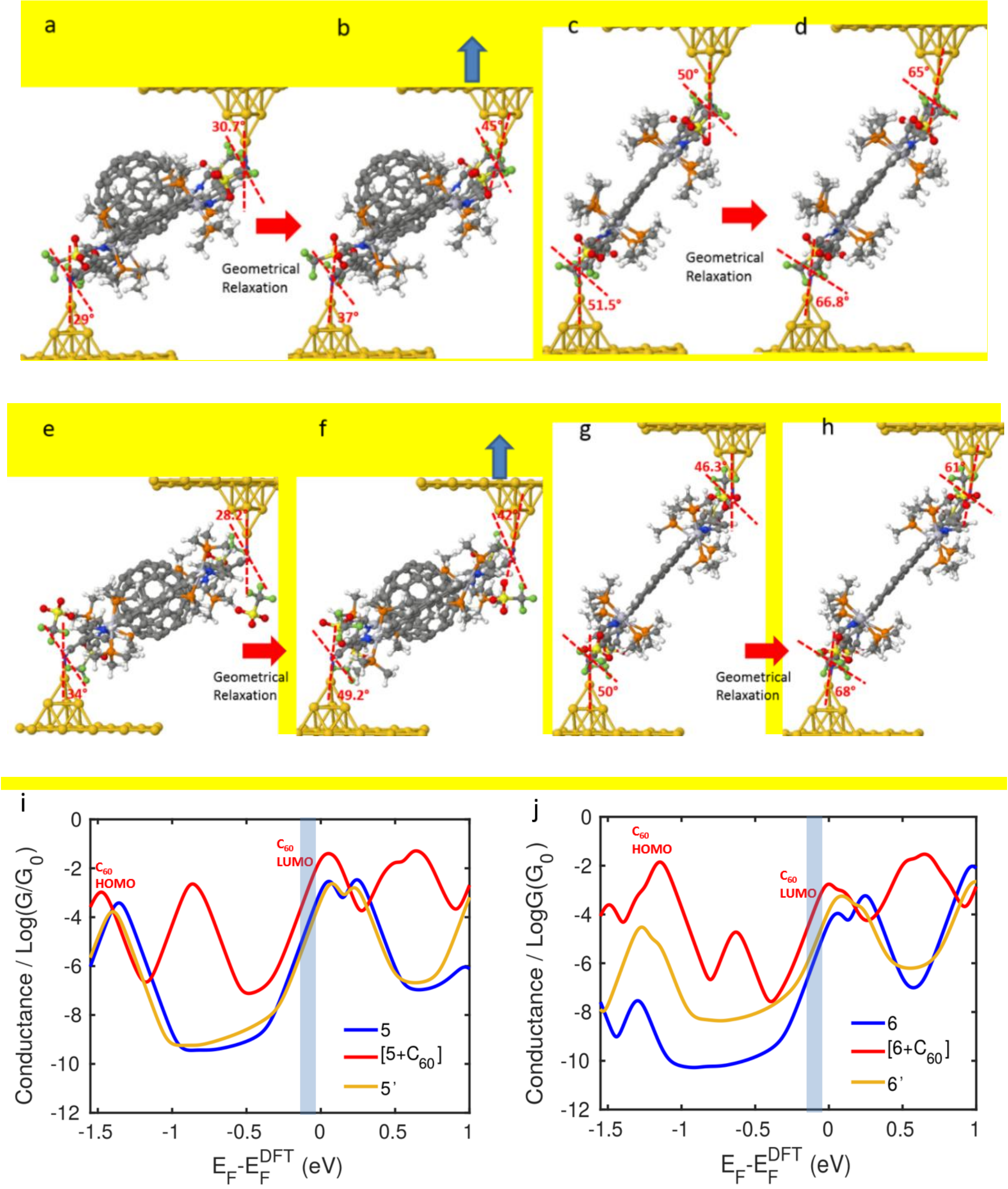

Fig. S42 Schematics of gold/molecule/gold break junctions during the pulling process and corresponding transport properties. a. Initial structure corresponding to high conductance of [**5**+C_{60}]. b. Optimal structure after geometrical relaxation. c. Initial structure of **5** or [**5**+C_{60}] after C_{60} falls out. d. Optimal structure after geometrical relaxation for structure c. e. Initial structure corresponding to high conductance of [**6**+C_{60}]. f. Optimal structure after geometrical relaxation. g. Initial structure of **6** or [**6**+C_{60}] after C_{60} falls out. h. Optimal structure after geometrical relaxation for structure g. i. Corresponding conductances as the function of fermi level relative to that given by DFT for **5**, [**5**+C_{60}], **5’** which is obtained from the optimal junction (shown in b) of [**5**+C_{60}] by

removing C_{60}. j. Corresponding conductances as the function of fermi level relative to that given by DFT for **6**, [**6**+C_{60}], **6'** which is obtained from the optimal junction (shown in f) of [**6**+C_{60}] by removing C_{60}.

energy window	-1.6~ -1.3eV		0 ~ 0.4eV	
**5**	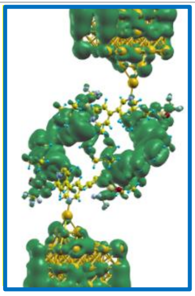		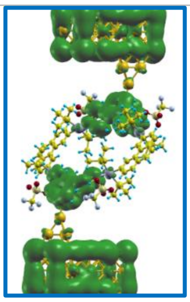	
[**5**+C_{60}]	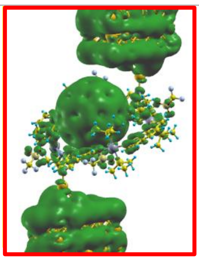	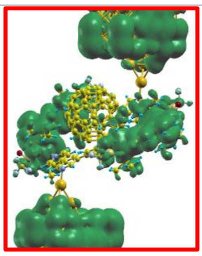	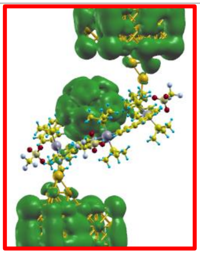	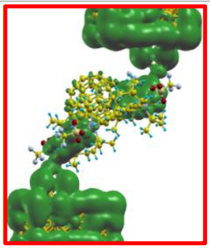
energy window	-1.6 ~ -1.4eV<br>C_{60} HOMO	-1. ~ -0.7eV<br>Shifted **5**<br>HOMO	0 ~ 0.3eV<br>C_{60} LUMO	0.3 ~1.0eV<br>Shifted **5**<br>LUMO

Fig. S43. Local Density of States (LDOS) of the junctions within different energy windows for tilted **5** and [**5**+C_{60}] in gold/molecule/gold junctions (Fig. S42d, b).

Energy window	-1.4 ~ -1.0 eV		0 ~ 0.5eV	
**6**	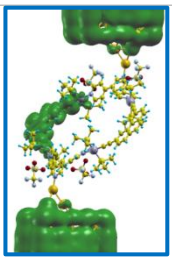		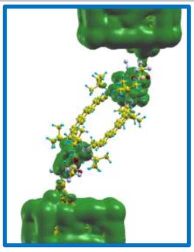	

[**6**+C_{60}]	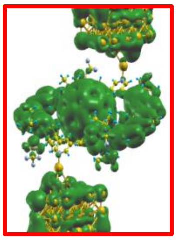	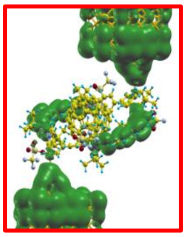	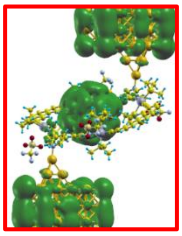	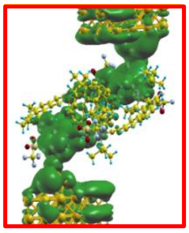
energy window	-1.6 ~ -1.3eV C_{60} HOMO	-0.8 ~ -0.5eV Shifted **6** HOMO	0 ~ 0.2eV C_{60} LUMO	0.5 ~ 0.8eV Shifted **6** LUMO

Fig. S44. Local Density of States (LDOS) of the junctions within different energy windows for tilted **6** and [**6**+C_{60}] in gold/molecule/gold junctions (Fig. S42h, f).

Table S5. Net charges of isolated complex **5**, [**5**+C_{60}], **6**, [**6**+C_{60}] and corresponding tilted junctions (Fig. S42d, b and h, f).

	**ISOLATED COMPLEXES**		**JUNCTIONS**	
**5**	0		-0.2	
[**5**+C_{60}]	0		-0.74	
	**5**: -0.23	C_{60}: 0.23	**5**: -0.18	C_{60}: -0.56
**6**	0		-0.16	
[**6**+C_{60}]	0		-0.80	
	**6**: -0.16	C_{60}: 0.16	**6**: -0.17	C_{60}: -0.63

The following figures are moved to Supplementary information from main manuscript.

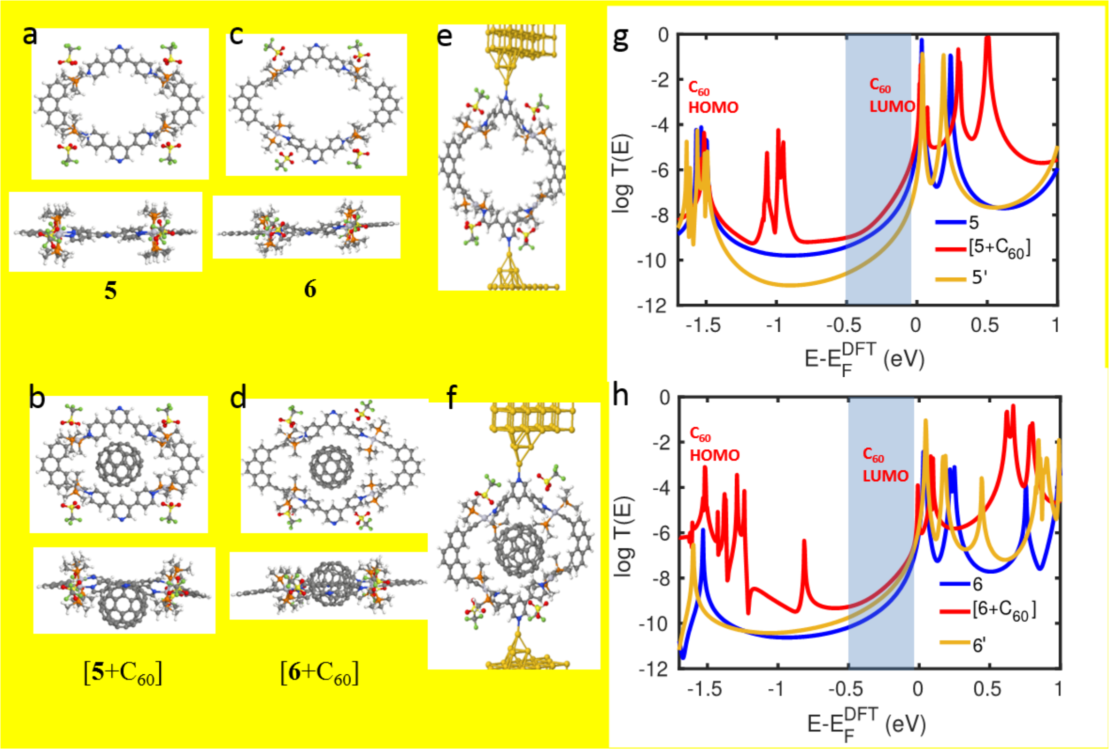

Fig. S33. Gold/molecule/gold junctions quantum calculations. Top and lateral views of metallocycle cation with -OTF anions (CF_3SO_3-). a for **5**, b for [**5**+C_{60}], c for **6**, d for [**6**+C_{60}]. Single-molecule junctions containing CF_3SO_3- anions, where the terminal atom of the gold tip is bonded above and below to the nitrogen atom of the pyridine with a Au-N distance of 2.3 Å. e for **6**, f for [**6**+C_{60}]. Gold atoms in the electrodes are coloured yellow. In the molecule: grey, blue, white represent carbon, nitrogen, and hydrogen atoms; green, light yellow, red, large grep, orange represent fluorine, sulphur, oxygen, platinum, phosphorus atoms. g, Transmission functions of **5**, [**5**+C_{60}] and the distorted **5** (denoted as **5'**) which is obtained from the junction of [**5**+C_{60}] by removing C_{60}. h, Transmission functions of **6**, [**6**+C_{60}] and the distorted **6** (denoted as **6'**) which is obtained from the junction of [**6**+C_{60}] by removing C_{60}.

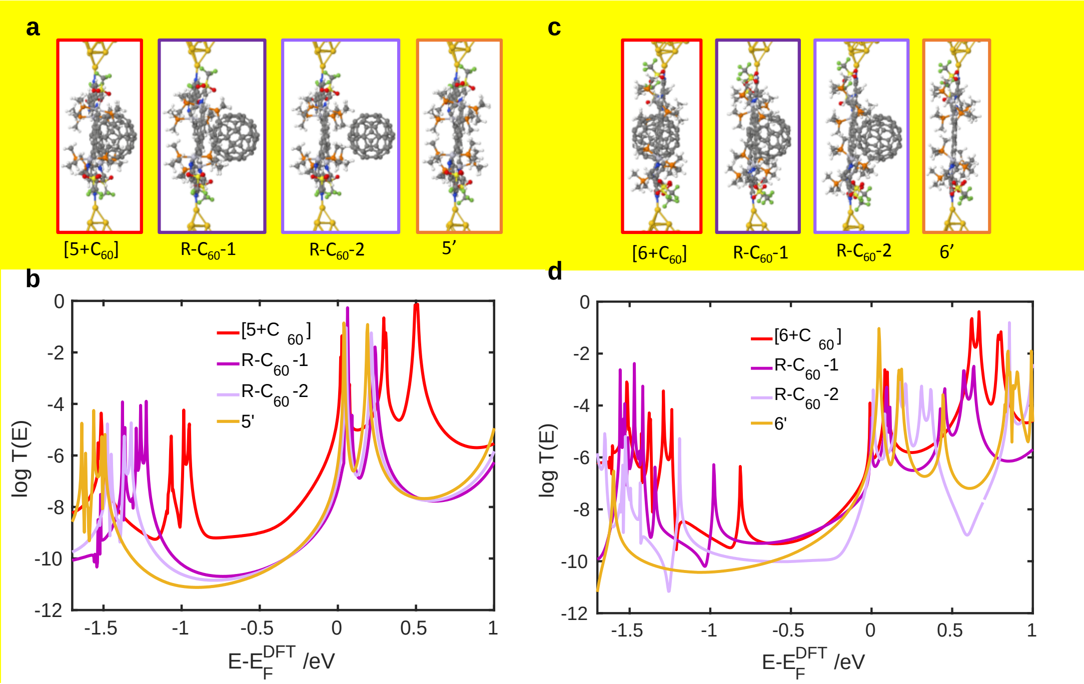

Fig. S34. Configurations and transmission functions corresponding to four stages during the release of C_{60}. a, Configurations and b, transmission functions of a series of [**5**+C_{60}] junctions. c, Configurations and d, transmission functions of a series of [**6**+C_{60}] junctions. For the configurations shown in the red, purple, light purple and yellow rectangular boxes, the corresponding transmission curves are shown red, purple, light purple and yellow plots respectively. The red and yellow transmission curves are the same with those shown in Fig. S33.

Reviewers' Comments:

Reviewer #2:
Remarks to the Author:
In the second revision the 2D histograms are a good addition and give important information about the distance at which the junction breaks. These new data have led to a significant further revision of the theory section taking account of revised junction geometry and tilting angles. The AFM scratching could have been more rigorously analyzed by using a statistical depth profiling to compare all points in scratched pit with surrounding unscratched area: this would have been more convincing than the noisy single lines shown. Otherwise, the experimental additions are acceptable and enhance the study.

Reviewer #3:
Remarks to the Author:
The authors have now answered my comments and questions and I can recommend publication.

In their reply the authors discuss several references and explain the importance of dispersion forces in binding energy of pyridine. Dispersion forces are not included in the present study but they should increase the values calculated here (with LDA).

Also there are several new paragraphs discussing the distortion of the host and of the contact angles. So some new knowledge is provided and the authors did not just repeat literature studies of binding energies.

The binding energies presented in this latest version show that the proposed geometries are stable. The relevance of these geometries is now established.

REVIEWERS' COMMENTS:

Reviewer #2 (Remarks to the Author):

In the second revision the 2D histograms are a good addition and give important information about the distance at which the junction breaks. These new data have led to a significant further revision of the theory section taking account of revised junction geometry and tilting angles. The AFM scratching could have been more rigorously analyzed by using a statistical depth profiling to compare all points in scratched pit with surrounding unscratched area: this would have been more convincing than the noisy single lines shown. Otherwise, the experimental additions are acceptable and enhance the study.

Reply: We thank the reviewer for this suggestion. We have re-determined the average heights of the films on Au(111) using six lines. The obtained values, of an averaged height of 1.2 nm and 1.3 nm for **5** and **6**, respectively, are similar as our previous results..

Reviewer #3 (Remarks to the Author):

The authors have now answered my comments and questions and I can recommend publication.

In their reply the authors discuss several references and explain the importance of dispersion forces in binding energy of pyridine. Dispersion forces are not included in the present study but they should increase the values calculated here (with LDA).

Also there are several new paragraphs discussing the distortion of the host and of the contact angles. So some new knowledge is provided and the authors did not just repeat literature studies of binding energies.

The binding energies presented in this latest version show that the proposed geometries are stable. The relevance of these geometries is now established.

Reply: We thank the reviewer for this recommendation of publication our manuscript.
